# Correction: Time of day is associated with paradoxical reductions in global signal fluctuation and functional connectivity

**DOI:** 10.1371/journal.pbio.3001258

**Published:** 2021-05-18

**Authors:** Csaba Orban, Ru Kong, Jingwei Li, Michael W. L. Chee, B. T. Thomas Yeo

There is an error in Figs 4 and 6, S5 and S7. The colours of two labels (Limbic A & Limbic B) are swapped in the legends of the cortical atlas (see Fig 4). Limbic A should refer to brain regions in the temporal lobes shown in the brighter colour, while Limbic B should refer to regions in the orbitofrontal cortex shown in the darker colour. The authors have provided corrected versions here.

**Fig 4:**
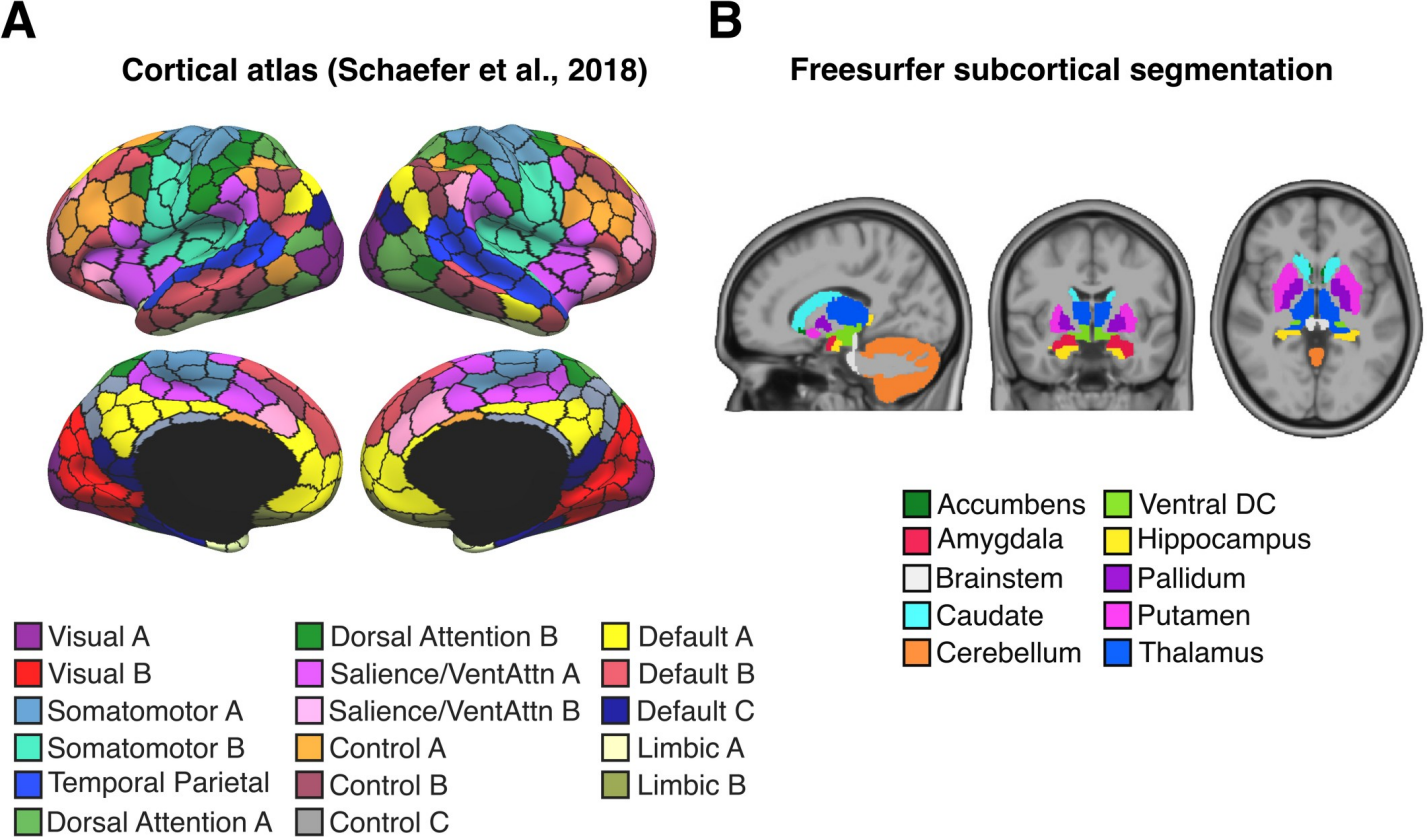
Four hundred nineteen ROIs. **(A)** Four hundred–area cortical parcellation in fs_LR surface space [45]. Parcel colours correspond to 17 large-scale networks [47]. *Image reproduced under a CC BY 4*.*0 license*, *credit*: *https*:*//doi*.*org/10*.*6084/m9*.*figshare*.*10062482*.*v1* **(B)** Nineteen subcortical regions defined in participant-level volume space [46]. *Image reproduced under a CC BY 4*.*0 license*, *credit*: *https*:*//doi*.*org/10*.*6084/m9*.*figshare*.*10063016*.*v1*. DC, diencephalon; ROI, region of interest; VentAttn, ventral attention.

**Fig 6:**
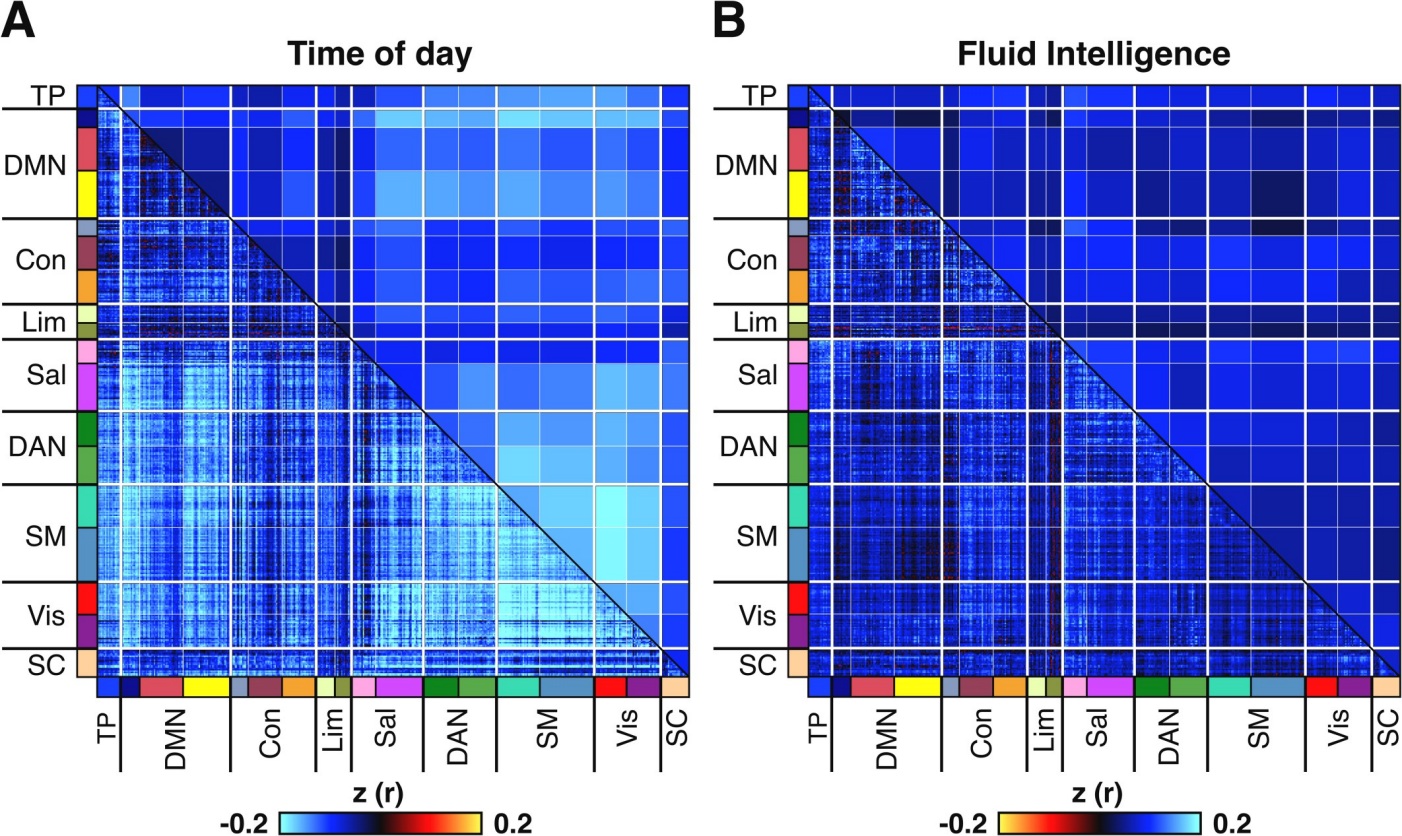
RSFC is negatively correlated with time of day across participants, with a magnitude surpassing the strength of correlation between fluid intelligence and RSFC. Results shown are for session 1 (see S5 Fig for session 2). **(A)** Correlation between time of day and RSFC across participants. **(B)** Correlation between fluid intelligence and RSFC across participants (with inverted colour scale to facilitate visual comparison with time of day effects). Colours in lower triangular of correlation matrix denote Pearson correlation coefficients. Colours in the upper triangular denote r values from the lower triangular averaged within network pairs. Colours on label axes denote correspondence of 419 regions to 17 large-scale cortical networks and to SC. Association between time of day and RSFC was significant in both sessions as assessed by network-based statistics (FDR-corrected at q < 0.05), whereas association between fluid intelligence and RSFC association was only significant in session 1 (FDR-corrected at q < 0.05). See Materials and methods for details of network-based statistics [51]. Fluid intelligence was chosen because it is a widely studied measure amongst resting-fMRI studies of brain-behavioural associations [50] and is one of the behavioural measures that is best predicted by resting fMRI [37,38,52]. Con, control network; DAN, dorsal attention network; DMN, default mode network; FDR, false discovery rate; fMRI, functional MRI; Lim, limbic network; RSFC, resting-state functional connectivity; Sal, salience network; SC, subcortical network; SM, somatomotor network; TP, temporal parietal network; Vis, visual network.

## Supporting information

S5 Fig RSFC region is negatively correlated with time of day across participants in session 2 (*n* = 865), with a magnitude that surpasses the strength of correlation between fluid intelligence and RSFC. (A) Correlation between time of day and RSFC across participants. (B) Correlation between fluid intelligence and RSFC across participants. Colours in lower triangular of correlation matrix denote z-transformed Pearson r correlation coefficients. Colours in the upper triangular denote z-transformed r values from the lower triangular averaged within network pairs. Colours on label axes denote correspondence of 419 regions to 17 large-scale cortical networks and to SC. Median absolute z values computed over the lower triangular were higher for time of day (0.13) than for fluid intelligence (0.04). Time of day–RSFC effects were significant, whereas RSFC–fluid intelligence effects were not significant for session 2, as assessed by network-based statistics (FDR-corrected at q < 0.05). Note that the colour scale for the fluid intelligence–RSFC effects was inverted to facilitate visual comparison with time of day effects. For session 1 results, see Fig 6 in the main text. FDR, false discovery rate; RSFC, resting state functional connectivity; SC, subcortex.
(TIF)

S7 Fig Global signal regression reduces magnitude of negative correlations between RSFC and time of day while introducing positive correlations for several large-scale circuits in both session 1 (*N* = 937) and in session 2 (*N* = 865). (A) Correlation between time of day and RSFC across participants. (B) Correlation between fluid intelligence and RSFC across participants. Levels of correlation are visibly stronger between time of day and RSFC than between fluid intelligence and RSFC. Colours in lower triangular of correlation matrix denote z-transformed Pearson r correlation coefficients. Colours in the upper triangular denote z values from the lower triangular averaged within network pairs. Colours on label axes denote correspondence of 419 regions to 17 large-scale cortical networks and to SC. Time of day–RSFC effects were significant in both sessions as assessed by network-based statistics (FDR-corrected at q < 0.05), whereas fluid intelligence–RSFC effects were significant only in session 1. FDR, false discovery rate; RSFC, resting-state functional connectivity; SC, subcortex.
(TIF)
