# Supplementary figures and images for: Time of day is associated with paradoxical reductions in global signal fluctuation and functional connectivity
Source: PLoS Biol. 2020 Feb 18;18(2):e3000602. doi: 10.1371/journal.pbio.3000602 (PMC7028250; doi:10.1371/journal.pbio.3000602)

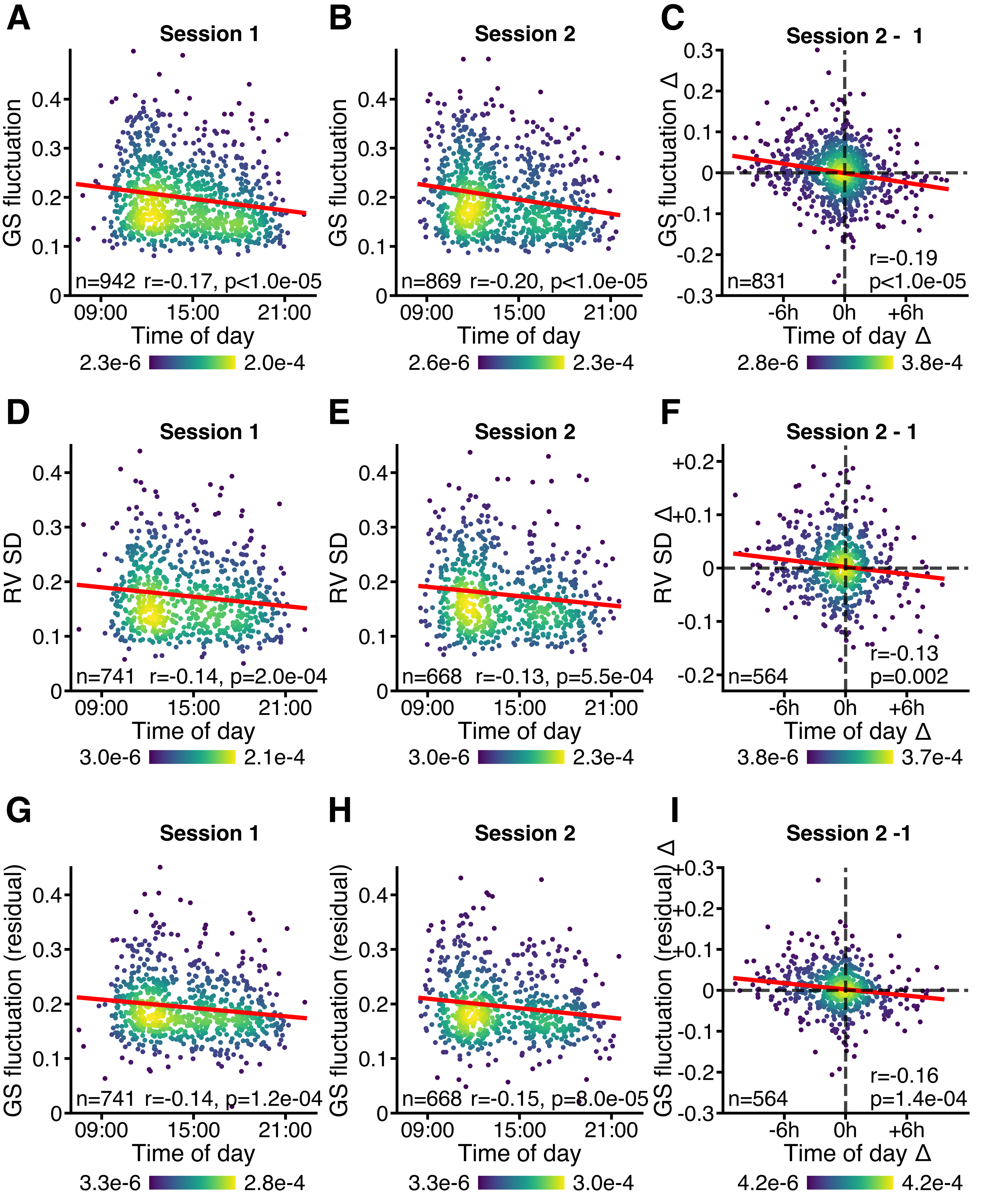

Supplement: S1 Fig — Scatterplots showing (A-C) effects of time of day on GS fluctuation, (D-E) effects of time of day on RV SD, and (G-I) effects of time of day on GS fluctuation after controlling for respiratory variation with colour-coding of data-point density. High density of data points around 12:30 PM is consistent with the planned timing of resting-state scans based on the HCP study protocol (HCP Reference Manual—1200 Subjects Release; Page 33). These same results are presented and described in more detail in Figs 1, 3 and S3, S4, S8 and S9 without colour-coding of data-point density. See S1 Data for underlying data. GS, global signal; HCP, Human Connectome Project; RV SD, standard deviation of respiratory variation. (TIF) [file pbio.3000602.s001.tif]

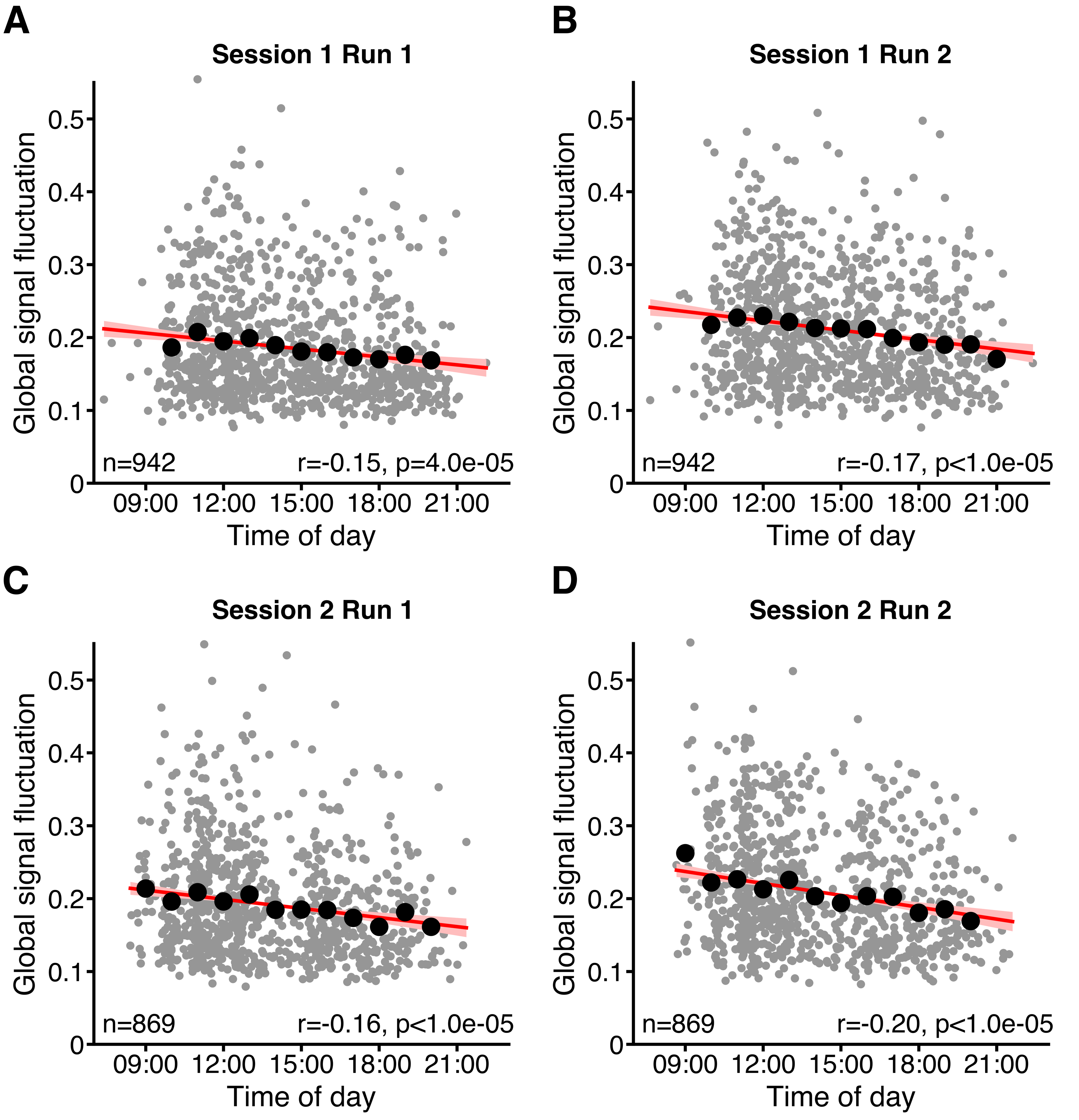

Supplement: S2 Fig — (A-D) Grey dots denote individual participants. Black dots show mean of GS fluctuation in hourly time windows. Line of best fit (red) was calculated based on data from all participants in each plot. Confidence interval is shown in light red. R values denote Pearson r correlation coefficient. p-Values were derived from 100,000 permutations while keeping family structure intact. GS fluctuation was defined as the standard deviation of the GS. See S2 Data for underlying data. GS, global signal; RV SD, standard deviation of respiratory variation. (TIF) [file pbio.3000602.s002.tif]

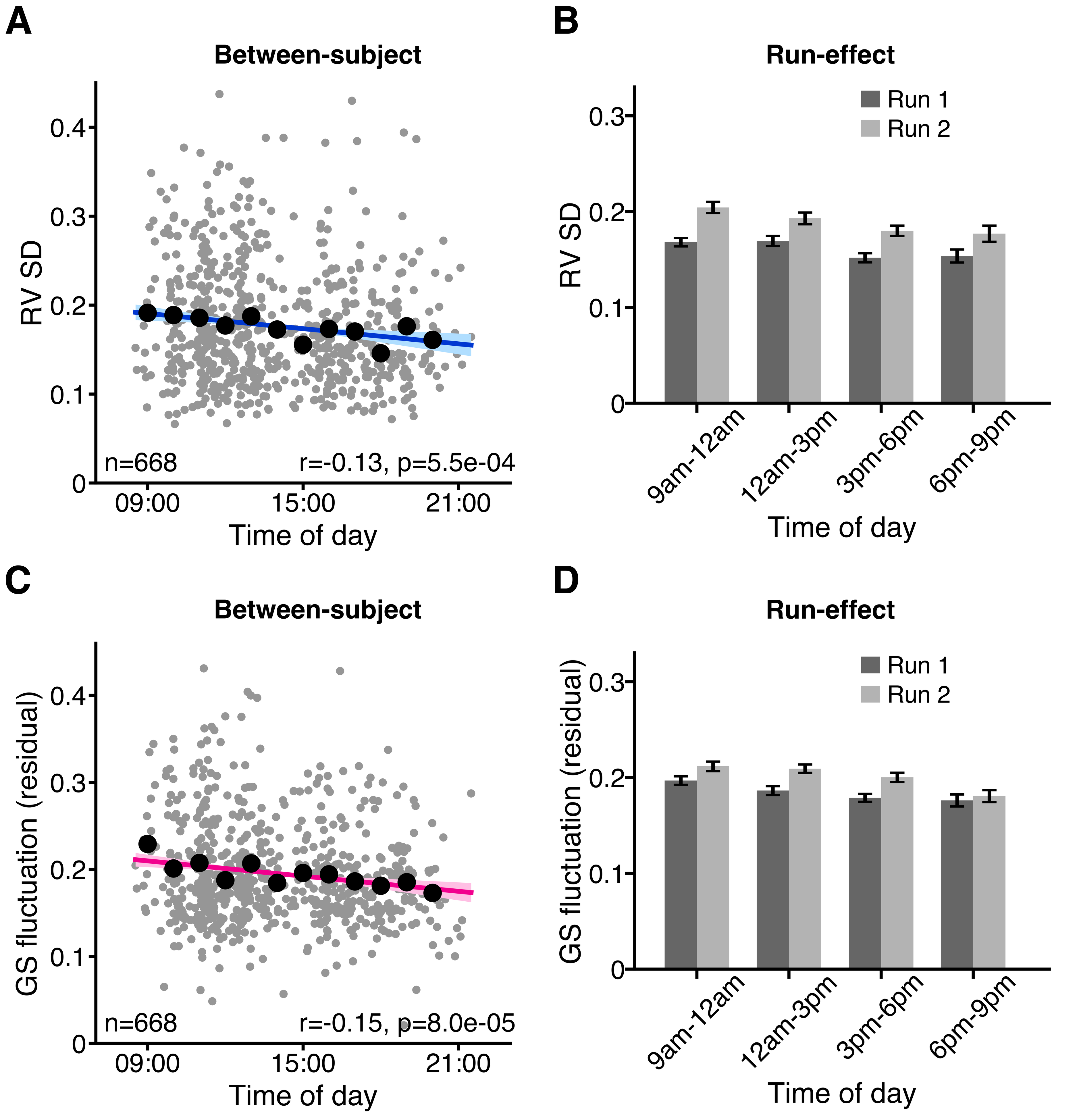

Supplement: S3 Fig — (A) Between-participant variation of RV SD. (B) Run effects on RV SD at different times of day. (C) Between-participant variation of GS fluctuation residual as a function of time of day. (D) Run effects on GS fluctuation residual at different times of day. GS fluctuation residual was computed by group-level regression of RV SD from GS fluctuation. Grey dots denote individual participants. Black dots denote mean of GS fluctuation in hourly (left) or 3-hourly time windows (centre). Confidence interval is shown in light blue or light pink. R values denote Pearson r correlation coefficients. p-Values were derived from 100,000 permutations while keeping family structure intact. See S1 Data for underlying data. GS, global signal; RV SD, standard deviation of respiratory variation. (TIF) [file pbio.3000602.s003.tif]

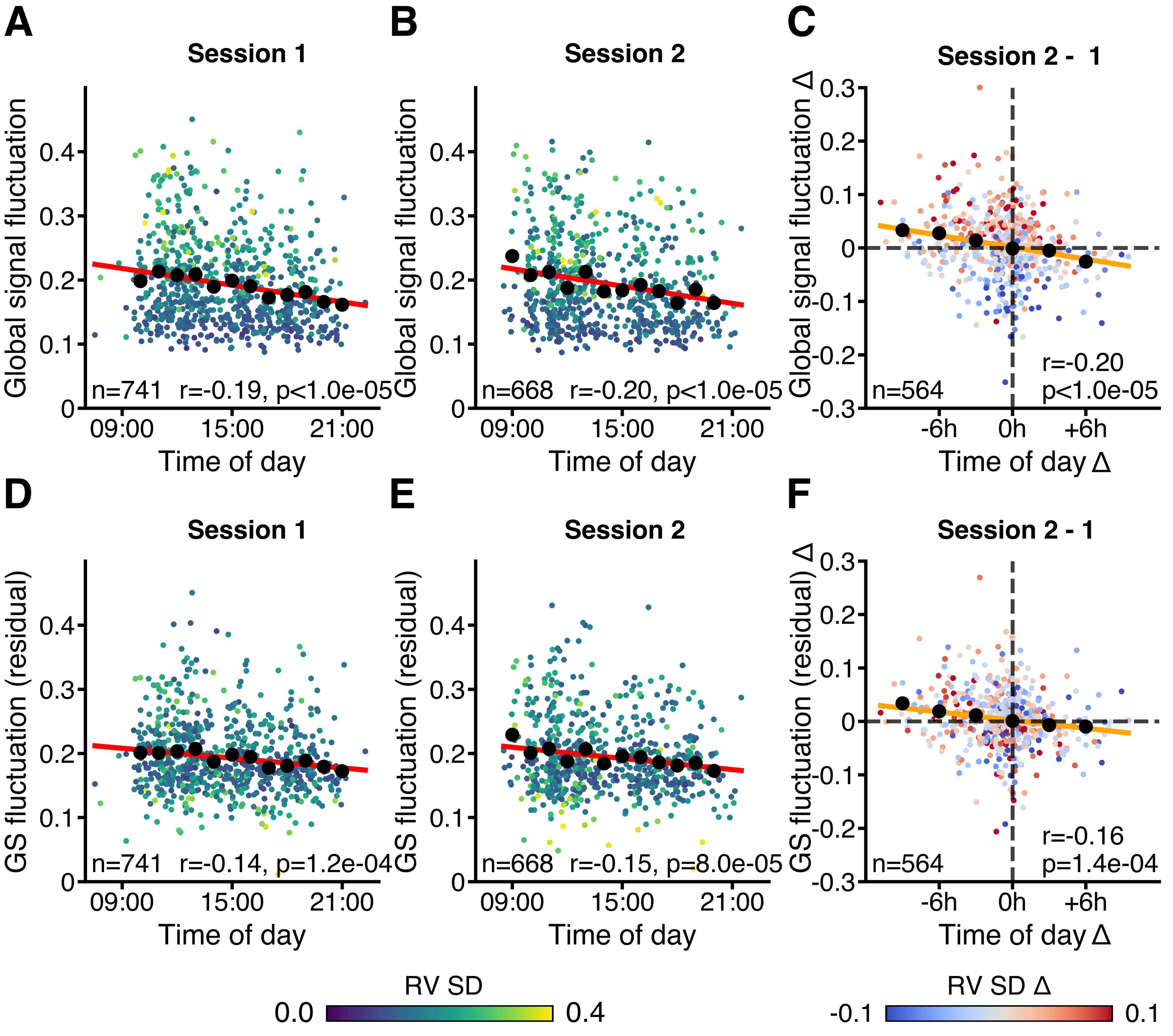

Supplement: S4 Fig — (A, B) Participants with greater RV SD (brighter dots) and scanned earlier in the day are more likely to exhibit greater GS fluctuation than those with lower RV SD (darker dots) and those scanned later in the day (C) Participants scanned a longer duration apart on the two sessions (greater time of day Δ) are more likely to exhibit a greater between-session difference in GS fluctuation (greater GS fluctuation Δ) and in RV SD (greater RV SD Δ). Participants with greater RV SD on session 2 are denoted in red, whereas those with higher RV SD on session 1 are denoted in blue. (D-F) As expected, statistically controlling for the effects of RV SD on GS fluctuation via group-level regression eliminates the apparent visual gradient pattern along the y-axis, reflecting the systematic contribution of RV SD to GS fluctuation. These results are also presented in Fig 3 and S2 Fig without colour coding of RV SD. See S1 Data for underlying data. GS, global signal; RV SD, standard deviation of respiratory variation. (TIF) [file pbio.3000602.s004.tif]

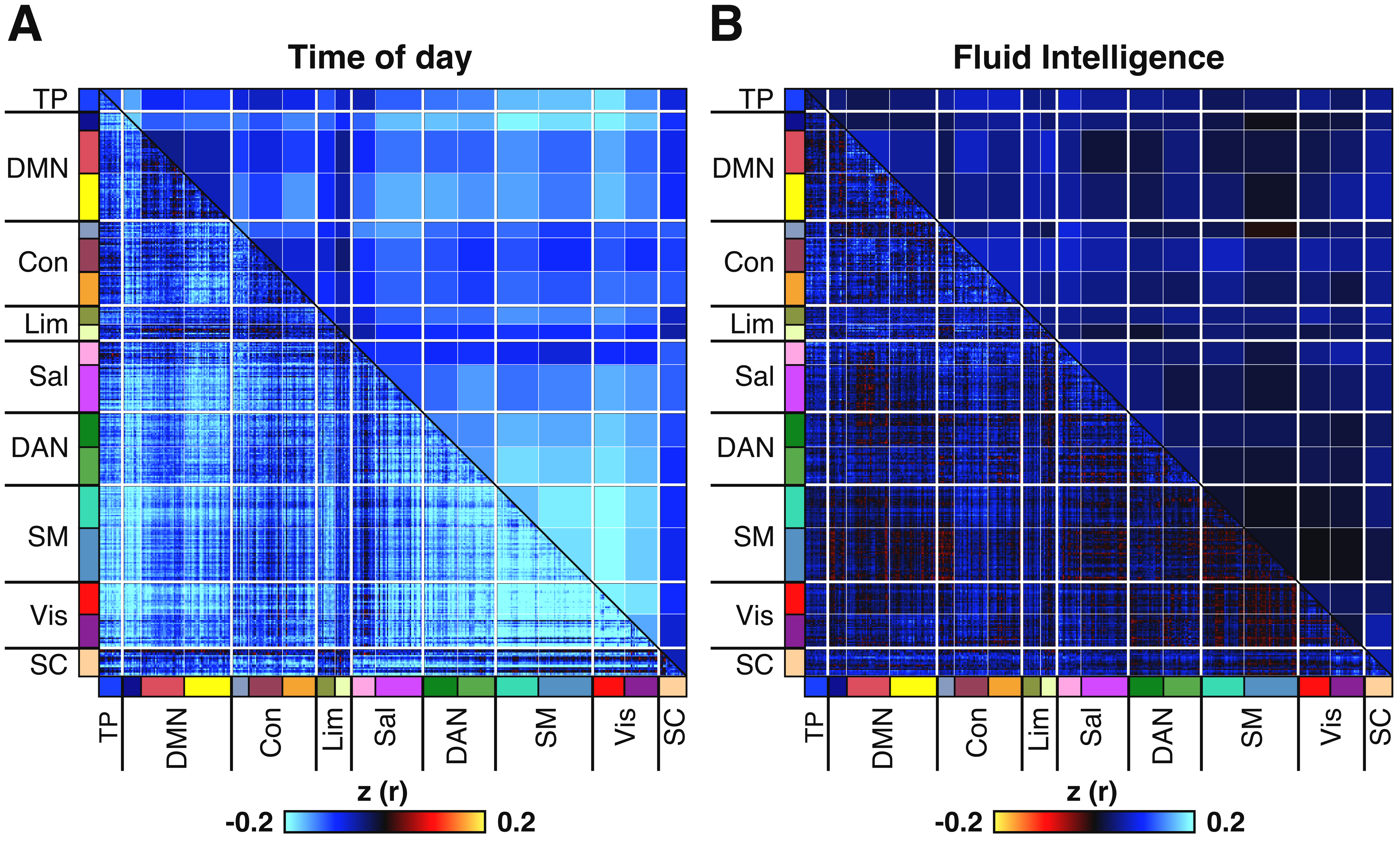

Supplement: S5 Fig — (A) Correlation between time of day and RSFC across participants. (B) Correlation between fluid intelligence and RSFC across participants. Colours in lower triangular of correlation matrix denote z-transformed Pearson r correlation coefficients. Colours in the upper triangular denote z-transformed r values from the lower triangular averaged within network pairs. Colours on label axes denote correspondence of 419 regions to 17 large-scale cortical networks and to SC. Median absolute z values computed over the lower triangular were higher for time of day (0.13) than for fluid intelligence (0.04). Time of day–RSFC effects were significant, whereas RSFC–fluid intelligence effects were not significant for session 2, as assessed by network-based statistics (FDR-corrected at q < 0.05). Note that the colour scale for the fluid intelligence–RSFC effects was inverted to facilitate visual comparison with time of day effects. For session 1 results, see Fig 6 in the main text. FDR, false discovery rate; RSFC, resting state functional connectivity; SC, subcortex. (TIF) [file pbio.3000602.s005.tif]

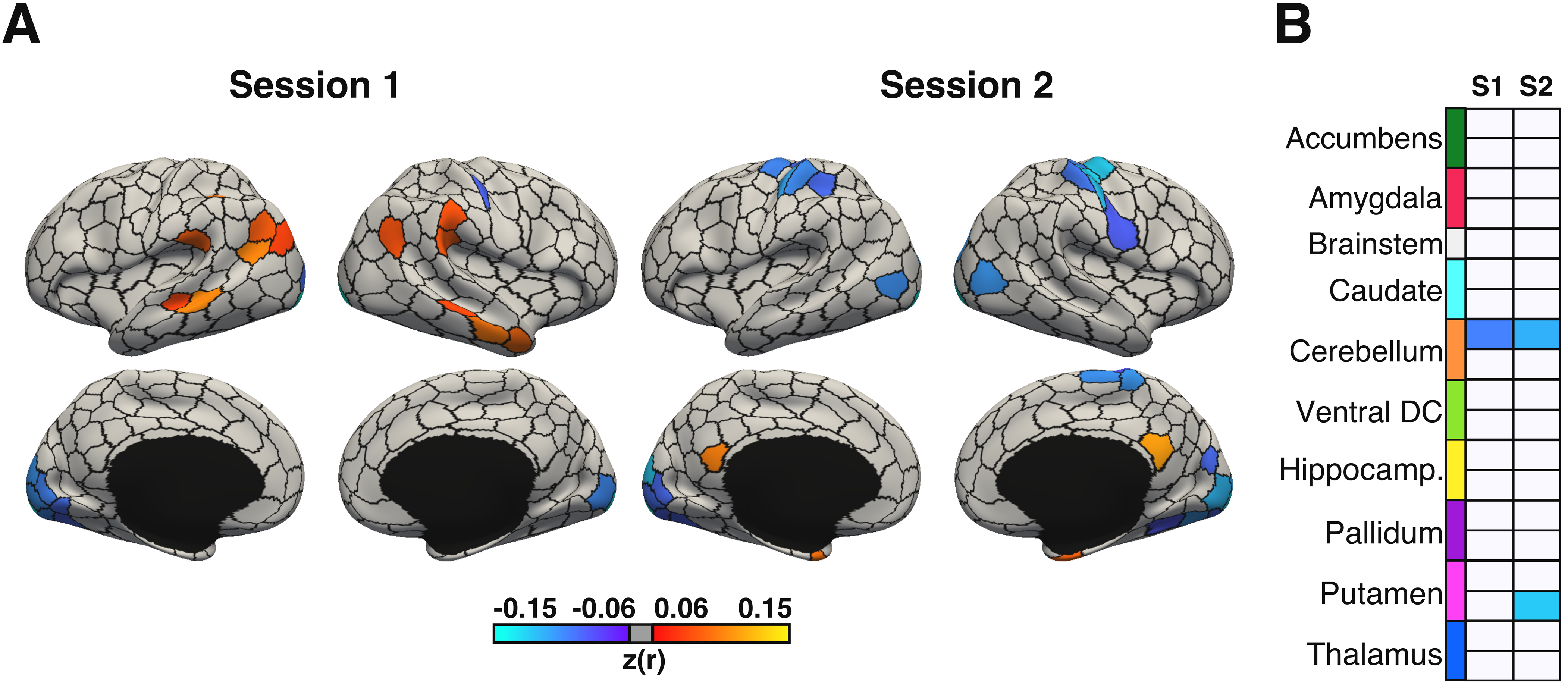

Supplement: S6 Fig — (A) Cortical regions showing significant correlations between time of day and BOLD signal fluctuation across participants in S1 (n = 942) and S2 (n = 869). (B) Subcortical regions showing significant correlations time of day and BOLD signal fluctuation across participants in S1 and S2. With the exception of brainstem, all subcortical regions are bilateral and presented as left-to-right hemisphere pairs (top to bottom). p-Values were derived from 100,000 permutations while keeping family structure intact. Colours (cool–warm) denote cortical and subcortical brain regions with significant z transformed Pearson r coefficients (q < 0.05, FDR-corrected), whereas nonsignificant regions are shown in grey. BOLD, blood oxygen level–dependent; FDR, false discovery rate; S1, session 1; S2, session 2. (TIF) [file pbio.3000602.s006.tif]

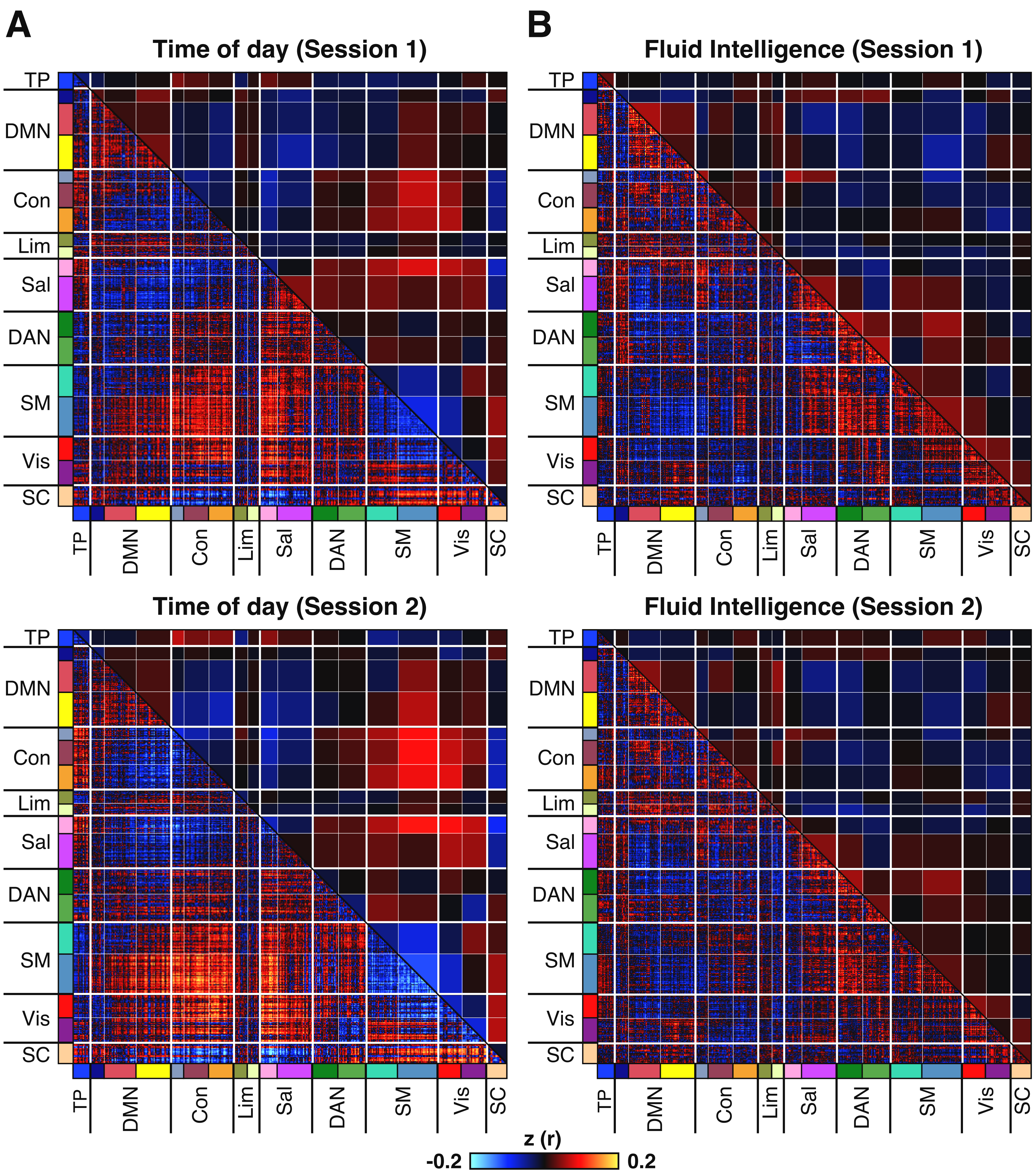

Supplement: S7 Fig — (A) Correlation between time of day and RSFC across participants. (B) Correlation between fluid intelligence and RSFC across participants. Levels of correlation are visibly stronger between time of day and RSFC than between fluid intelligence and RSFC. Colours in lower triangular of correlation matrix denote z-transformed Pearson r correlation coefficients. Colours in the upper triangular denote z values from the lower triangular averaged within network pairs. Colours on label axes denote correspondence of 419 regions to 17 large-scale cortical networks and to SC. Time of day–RSFC effects were significant in both sessions as assessed by network-based statistics (FDR-corrected at q < 0.05), whereas fluid intelligence–RSFC effects were significant only in session 1. FDR, false discovery rate; RSFC, resting-state functional connectivity; SC, subcortex. (TIF) [file pbio.3000602.s007.tif]

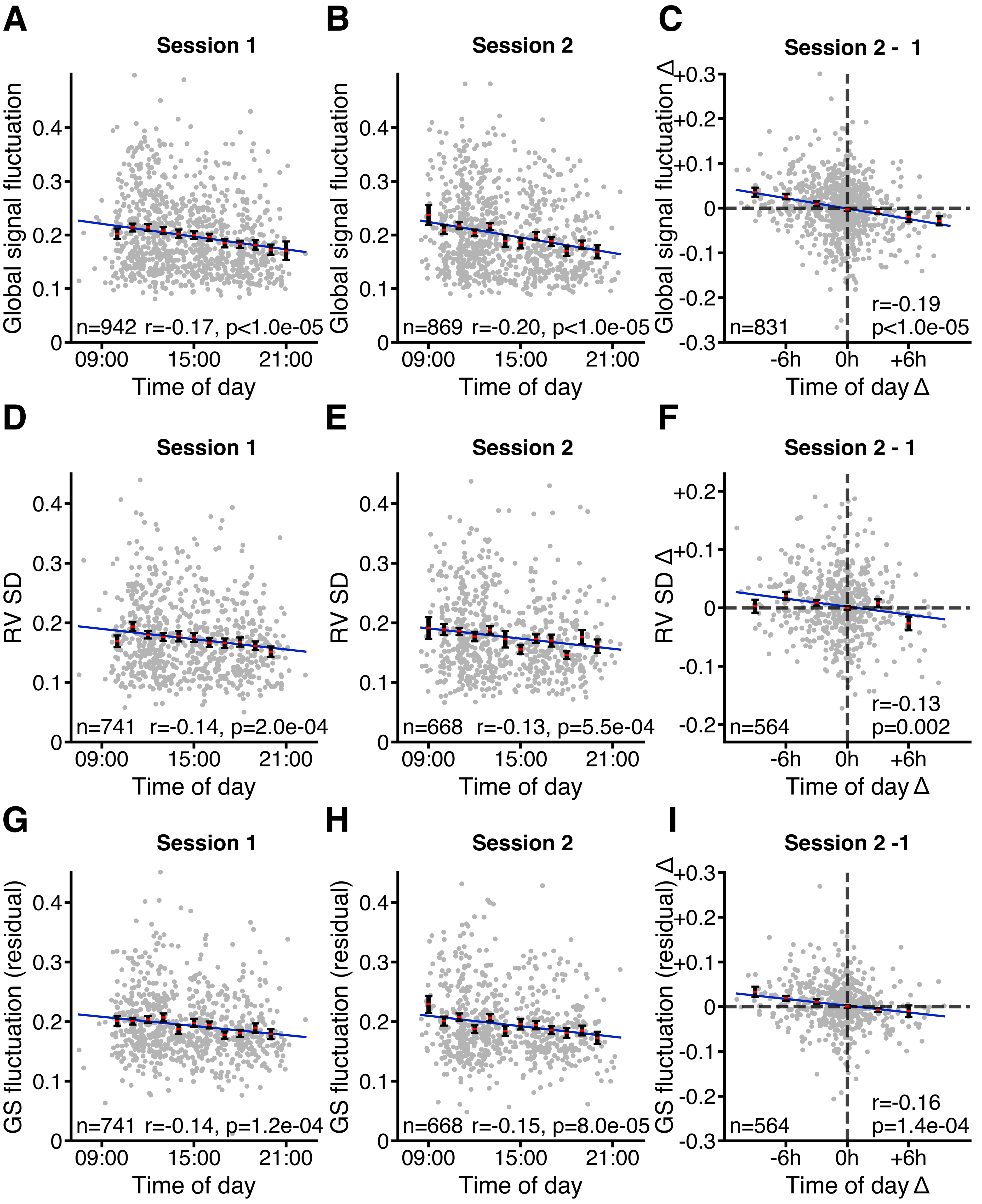

Supplement: S8 Fig — Scatterplots showing (A-C) effects of time of day on GS fluctuation, (D-E) effects of time of day on respiratory variation, and (G-I) effects of time of day on GS fluctuation after controlling for respiratory variation. Error bars show standard error of hourly windowed means. These scatterplots are presented and described in more detail in Figs 1, 3, S3, S4 and S9, without the windowed standard error bars. See S1 Data for underlying data. GS, global signal. (TIF) [file pbio.3000602.s008.tif]

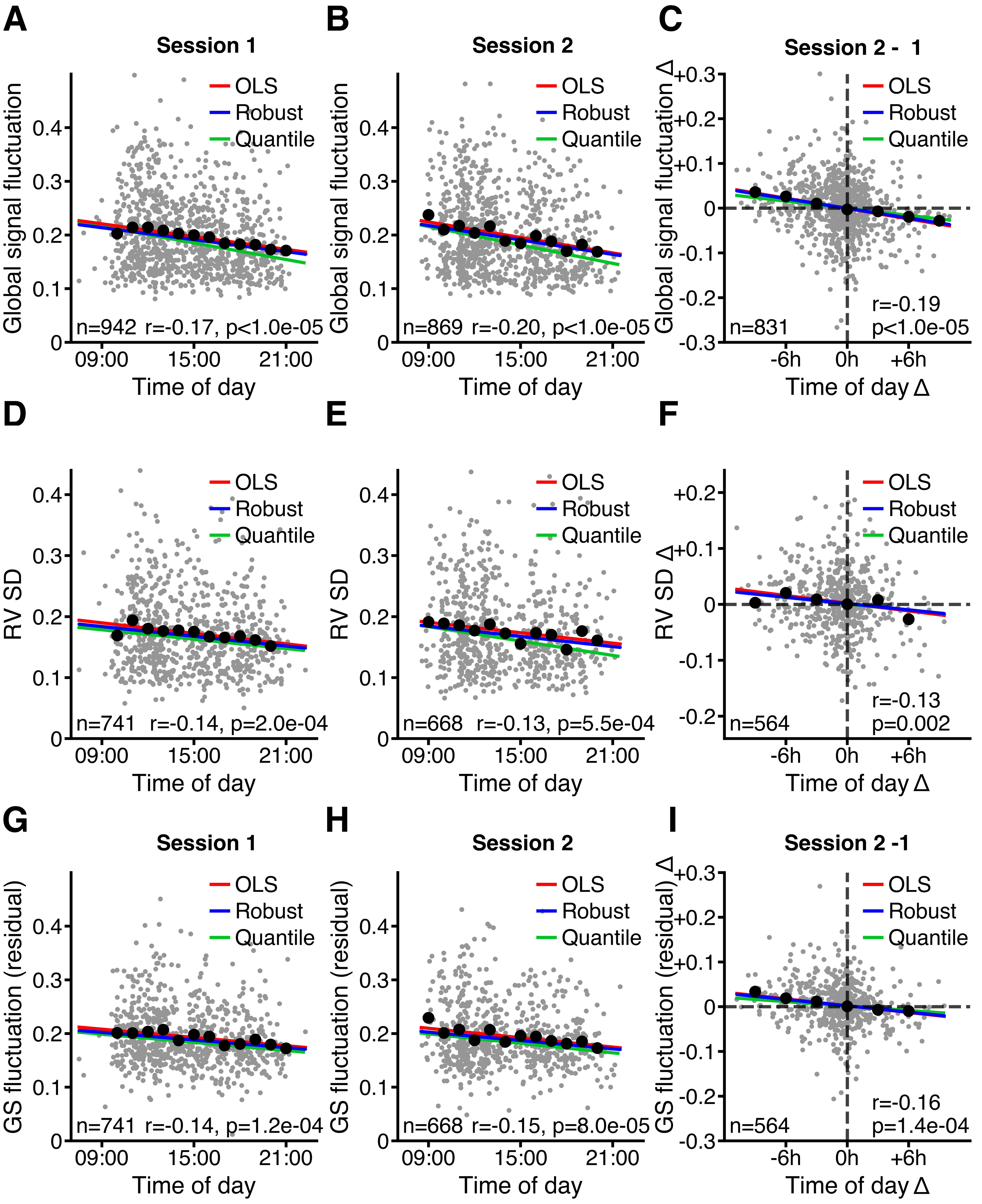

Supplement: S9 Fig — Scatterplots showing (A-C) effects of time of day on GS fluctuation, (D-E) effects of time of day on GS fluctuation on respiratory variation, and (G-I) effects of time of day on GS fluctuation on GS fluctuation after controlling for respiratory variation. Lines of best fit were computed using three different methods: OLS regression (red), robust regression (blue), and quantile regression (green). Robust regression and quantile regression were chosen because these two approaches are less susceptible to heteroscedasticity than OLS. The r and p-values shown are from the OLS regression. The same scatterplots are also presented in Figs 1, 3, S1, S3, S4 and S8. See S1 Data for underlying data. GS, global signal; OLS, ordinary least squares. (TIF) [file pbio.3000602.s009.tif]

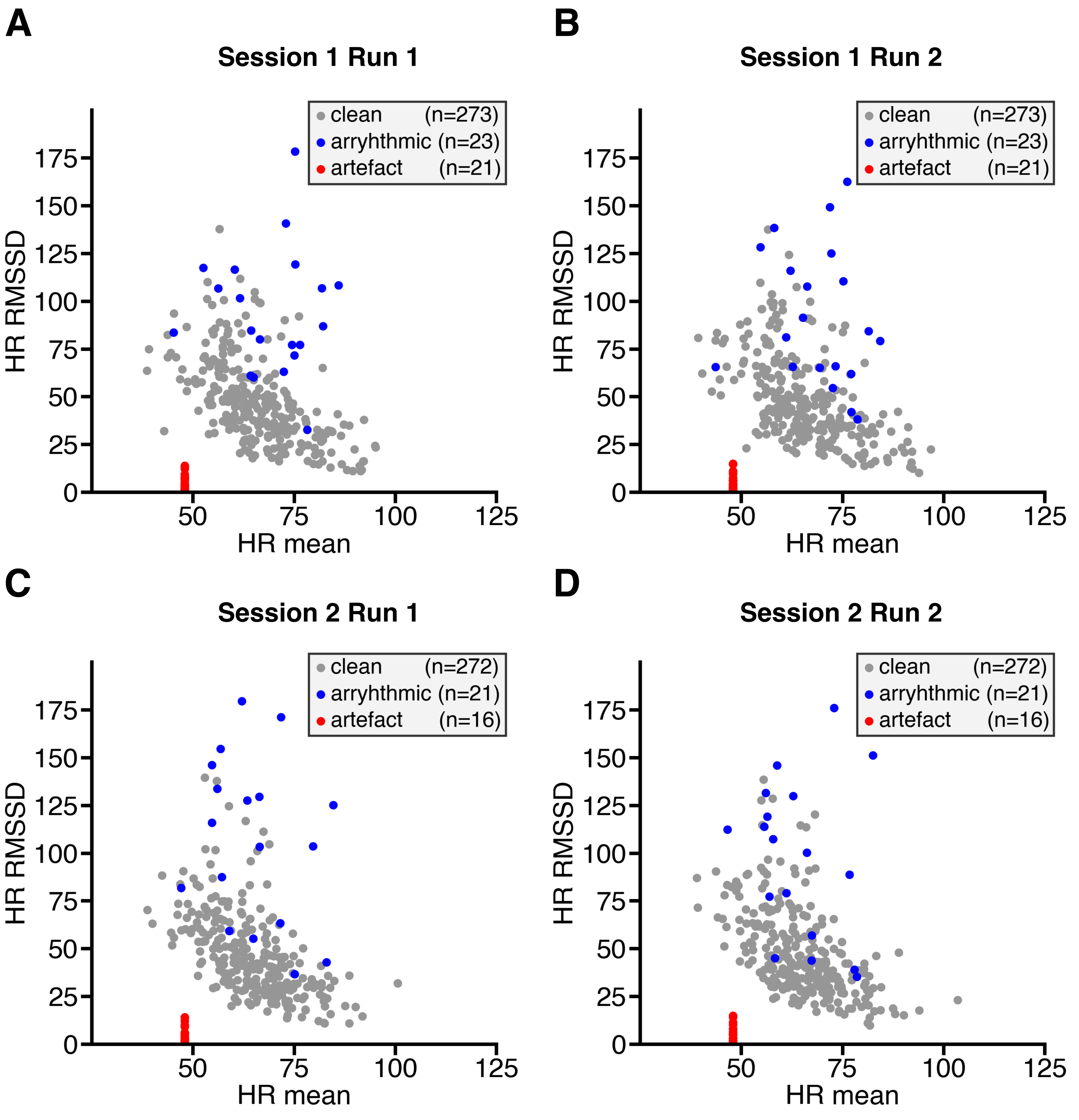

Supplement: S10 Fig — Data are shown for participants with pulse oximetry traces with sufficient quality to enable reliable peak detection. For a participant to be included in session-level analyses, both runs had to pass quality criteria. Despite good-quality peak detection in these participants, there were some additional anomalies. In several runs, participants exhibited an HR of exactly 48 beats per minute, with very low levels of HRV, which were interpreted as likely artefactual in origin (shown in red). Other participants (shown in blue) were identified as likely having arrhythmia of nonsinus origin based anomalous distributions on Poincaré plots (not shown). Some of these participants are not visible on the plot because they have very high HRV (>200 RMSSD). The remaining runs were deemed suitable for analysis of cardiac data (shown in grey). See S1 Data for underlying data. HR, heart rate; HRV, heart rate variability; RMSSD, root-mean-square of the successive differences. (TIF) [file pbio.3000602.s010.tif]
