# Supplementary material for: Time of day is associated with paradoxical reductions in global signal fluctuation and functional connectivity
Source: PLoS Biol. 2020 Feb 18;18(2):e3000602. doi: 10.1371/journal.pbio.3000602 (PMC7028250; doi:10.1371/journal.pbio.3000602)
Supplement: Attachment — Submitted filename: Orban_2019_revised_Reply_to_Reviewers.pdf [file pbio.3000602.s013.pdf]

Reply to reviewers PBIOLOGY-D-19-01472R1 "Time of day is associated with paradoxical reductions in global signal fluctuation and functional connectivity"

We thank the reviewers for their close read of this manuscript and insightful comments. Several important suggestions were made for improvement and we have considered each carefully and revised accordingly. Please find below detailed responses (in blue) to the reviewer comments (in italics). For convenience, changes to the manuscript are quoted verbatim (normal font) when appropriate. We believe the manuscript is much improved and hope it is now suitable for publication.

Reviewer #1:

R1Q1 General comments: Matthew Glasser. The authors present an interesting study of the effect of time of day scanned on global signal. I am not sure I understand the mechanism of the finding and it is not what I would have expected based on prior literature. Because the authors used HCP data and started from the recommended HCP preprocessing, their methods are generally of very high quality. I have a few minor comments. It will of course be important to replicate these findings after appropriate global noise removal, e.g. with temporal ICA, when that is available. I think it also would be interesting to extend the work to task fMRI data that has been appropriately cleaned for spatially specific and global noise to see if this pattern holds when subjects are performing a task. Additionally, it would be interesting to investigate which temporal ICA components are driving this phenomenon (I expect RC1 and/or RC5 from Glasser et al 2018 Neuroimage). If this is driven mainly by RC1, the phenomenon might not be seen in task fMRI data as RC1 is not present (and RC5 is much weaker). None of these data are yet publicly available for the authors to use; however.

We thank the reviewer for the positive comments. We agree with the reviewer that it will be interesting to perform a follow up study with temporal ICA components. We now discuss this point in the manuscript:

Section 4.11 of Discussion

> There are many avenues to extend the current study. For example, it will be interesting to explore whether the same effects can be see during task-fMRI. In addition, Glasser and colleagues proposed the use of temporal ICA (Glasser et al., 2018) to decompose the fMRI data into multiple components, some of which appeared to reflect “global” artefacts, which can then be more selectively removed. It would be interesting to investigate how these global artifactual components might relate to time of day. Furthermore, some “global” components are present only during resting-fMRI, but not task-fMRI (Glasser et al., 2018). Thus, some of the effects we observe in this study might not appear in task-fMRI.

(R1Q2) It sounds like the authors carried out the laborious task of QC of the HCP’s physiological noise measures. Ideally this data would be shared back to the HCP for public distribution as recommended in Glasser et al 2019 Neuroimage.

Yes, indeed, the first author manually quality checked the physio data from all four runs of the S1200 resting state fMRI dataset in order to select subjects with acceptable quality pulse and respiratory data. Alongside this manuscript we will post a list of these subjects on our lab's GitHub page, which we will also be happy to share with the HCP for public distribution. We are also in the process of producing a separate paper, in collaboration with Jonathan

Power, that will describe the QC of the HCP's physiological data more extensively, which we plan to submit in the near future.

(R1Q3) The use of a stringent FD threshold in HCP data is problematic for reasons discussed in Glasser et al 2018 Neuroimage and Power et al 2019 BioRXiv. A better motion measure would be dips and peaks after sICA+FIX cleanup in DVARS.

As per the reviewer's suggestion, we have now added DVARS dips/peaks to Figure 2 (replicated below for your convenience). Consistent with the other head motion measures, DVARS dips/peaks (%) showed a significant correlation across subjects with GS fluctuation, but not with time of day. We have updated the manuscript as follows:

Section 5.1.3 of Materials & Methods

> DVARS dips/peaks were defined for each subject as the percentage of frames which deviated by at least 75 from the median DVARS value (Glasser et al., 2018). Based on prior work we derived DVARS dips/peaks from the unstructured noise timeseries (Glasser et al., 2018). Unstructured noise timeseries were computed by regressing each ICA-FIX signal component from the ICA-FIX denoised image of each subject.

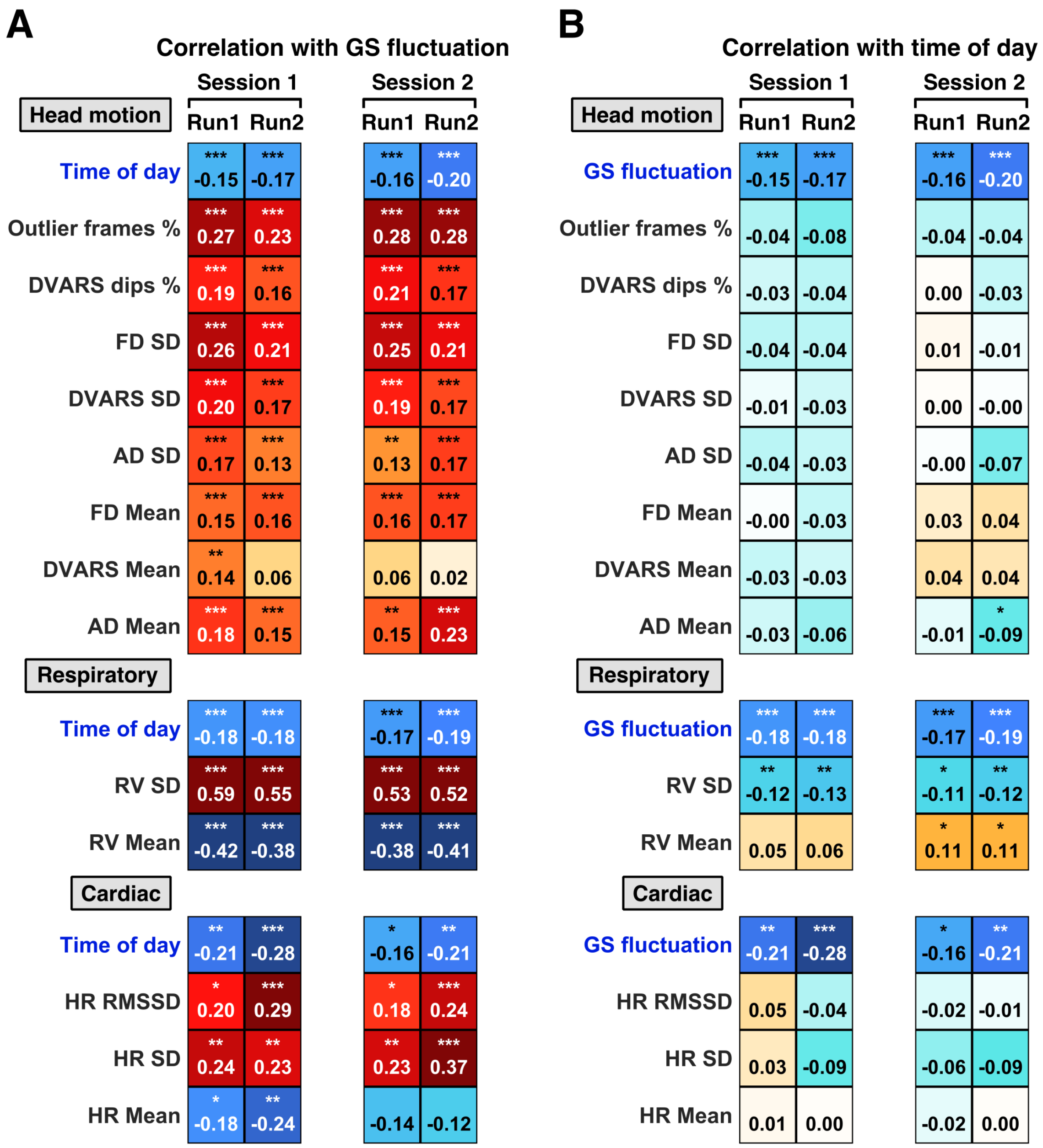

Figure 2. Head motion, respiratory and cardiac measures are strongly correlated with global signal (GS) fluctuation, yet only respiratory variation shows association with time of day. (A) Between-subject correlations of thirteen run-level summary metrics with GS fluctuation. **(B)** Between-subject correlations of thirteen run-level summary metrics with time of day. Due to exclusion of subjects with poor physiological data quality, different subgroups of subjects were used for analyses of head motion (Session 1: N = 942, Session 2: N = 869), respiratory (Session 1: N = 741, Session 2: N = 668) and cardiac measures (Session 1: N = 273, Session 2: N = 272). Correlation between GS fluctuation and time of day was repeated in each subgroup. Numbers denote z-scored Pearson r correlation coefficients. Stars indicate significant correlations following FDR correction (*q < 0.05; ** q < 0.01; *** q < 0.001). SD: standard deviation; AD: absolute displacement; FD: framewise displacement; RV: respiratory variation.

(R1Q4-Q5) The HCP structural acquisition was described in Glasser et al 2013 Neuroimage. The HCP structural preprocessing was a customized pipeline that included FreeSurfer along with other things.

We have inserted citations of Glasser et al., 2013 to Sections 5.1.2 and 5.1.3. In addition we have rephrased and extended the following paragraph to clarify that the structural preprocessing pipeline included more than just Freesurfer with additional citations of relevant papers:

Section 5.1.3 of Materials & Methods

> T1 structural images were preprocessed using a custom pipeline that utilised Freesurfer (Fischl, 2012) along with other software and algorithms (e.g. Glasser and Van Essen, 2011; Rilling et al., 2012; Marcus et al., 2013; Robinson et al., 2014). Preprocessing steps included brain extraction, subject-level volumetric segmentation of subcortical regions, cortical surface reconstruction and registration of each subject's cortical surface mesh to a common spherical coordinate space (Fischl et al., 1999a, 1999b). For a more detailed description of the structural preprocessing pipeline see Glasser et al., 2013.

(R1Q6) I wouldn't say "corrected for head-motion via a 24 parameter regression" as we know from Power's work among others that movement regressors hardly correct for subject head motion and even doing the 24 parameter movement regression is becoming more controversial given its potential to remove neural signal while contributing modest additional denoising above sICA+FIX (Glasser et al 2019 Neuroimage). Better to just say that the movement regressors were regressed out.

We thank the reviewer for highlighting this and have edited this part accordingly:

Section 5.1.4 of Materials & Methods

> The data was first de-trended with a temporal high-pass filter, followed by regression of head motion parameters, before undergoing denoising via spatial ICA-FIX (Griffanti et al., 2014; Salimi-Khorshidi et al., 2014).

Reviewer #2:

(R2Q1) General comments: This is study of the time of day on global signal fluctuations as well as regional fluctuations and connectivity is carried out on the Human Connectome Project dataset. The results are clear even though the authors fail to provide a compelling mechanism for this paradoxical effect. Changes in cortical excitability over the course of the day, at least to me, seems to be the most interesting explanation. The appropriate controls are performed although I believe that the nonlinear and spatially varying effects of respiration on GS may not be fully accounted for in their respiration effect normalization. In general, it is an interesting finding and a clear paper overall.

We thank the reviewer for the positive comments.

(R2Q2) In the abstract, the reference to a 1% to 3% BOLD signal change does not do much in putting a 22% decrease in context since the % BOLD change is of the raw signal and the 22% decrease is of a standard deviation measure. My guess is that the global standard deviation in terms of percent, shifted from 2% to 1.6%.

We thank the reviewer (as well as Reviewer 3) for challenging us on this point. We agree that raw signal in task and standard deviation change at rest are not comparable, so we have removed it from the abstract. We have also updated a sentence in the results, which now describes time of day related change in GS fluctuation in units of BOLD percent change.

Section 3.1 of Results

> From 9am to 9pm, the magnitude of GS fluctuation decreased from 0.22% of mean BOLD signal flucutation to 0.17% (on average) in both Sessions 1 and 2.

We have also added a description of how we computed BOLD percent change in the Methods section.

Section 5.2 of Materials & Methods

> Global signal fluctuation was always plotted in units of BOLD percent signal change. This was computed by dividing each subject's global signal fluctuation scores by 10,000 and multiplied by 100, given that each resting state fMRI run of each subject had originally undergone grand-mean scaling to a value of 10,000 during preprocessing.

(R2Q3) Global signal fluctuations also include respiration effects, CSF pulsation, and brain pulsation. It may very well be that these could be reduced with dehydration as the day progresses. You mention appropriately, the possibility of fluid intake at the end of your paper.

We thank the reviewer for drawing our attention to dehydration as a possible mechanism. We have added this to the discussion:

Section 4.8 of Discussion

> Time of day effects have not only been observed in studies of brain activity but also in studies of brain structure (Jiang et al., 2014; Elvsashagen et al., 2015; Nakamura et al., 2015; Trefler et al., 2016; Thomas et al., 2018; Karch et al., 2019). Notably, some studies have reported morning-to-evening reductions in total brain and/or grey-matter volume at rates comparable to age-related annual atrophy (Nakamura et al., 2015;

Karch et al., 2019). Hydration levels have been mentioned as a possible mechanism, given that hydration has been shown to modulate measurements of total brain volumes in some studies (Streitbürger et al., 2012; Nakamura et al., 2014), though not all (Karch et al., 2019). Dehydration could also potentially impact BOLD signal fluctuation by reducing the amplitude of brain (or CSF) pulsation, or the dynamic range of cardiac and respiratory activity. Unfortunately, fluid intake of participants was not tracked in the HCP protocol.

(R2Q4) It is observed that RV standard deviation was also correlated with time of day as well. It is mentioned that the correlation to time of day was a bit was weaker than that of GS std. Using linear regression to correct for this, the GS standard deviation effect remains. Linear regression to remove the effect may miss the nonlinear impact that respiration may have. It has been shown recently - in a talk at OHBM by Catie Chang - that the "respiration response function" first described by Birn et al actually varies considerably throughout the brain, so the respiration effect may still perhaps be having an impact on GS that is unable to be regressed out by simple linear modeling.

We thank the reviewer for highlighting this limitation, which we now discuss in more detail:

Section 4.4 of Discussion

Admittedly linear regression of RV SD from GS fluctuation might miss potential non-linear effects of respiration on GS fluctuation. Modelling of respiratory influences on BOLD signal fluctuations is non-trivial as respiratory response functions often fail to capture the impact of pauses and subtle variations in rate and depth of breathing (Birn et al., 2006; Power et al., 2017). Recently it has also been shown that resting state BOLD signal fluctuations that track respiratory dynamics exhibit spatially heterogeneous time-courses (lags and shape) throughout the brain (Chen et al., BioRxiv). Thus we cannot fully rule out respiratory mechanisms underpinning our observed time of day effects on BOLD signal fluctuation.

(R2Q5) On page 22 you mentioned: "These latter effects, however, might only become apparent once the global signal is removed from the data, for example, as was the case with the positive correlations between time of day and Somatomotor – Control network RSFC in the current study (Figure S5)" On the contrary, this might also be an artifact induced by GSR as Murphy et al argued. They showed that GSR induces artifactual correlations in resting state signals that were previously uncorrelated.

We agree with the reviewer's point and have added the following to the discussion:

Section 4.7 of Discussion

Alternatively, the positive correlations observed between time of day and Somatomotor – Control network RSFC might be artefacts introduced by GSR. Indeed, several studies have shown using simulations that GSR can introduce previously non-existent connectivity, bias short and long distance connectivity, and induce spurious group differences (Murphy et al., 2009; Saad et al., 2012; Satterthwaite et al., 2012). However, others have cautioned that these simulations may not generalise well to empirical data where the number of independent signals is substantially larger or where there is a large shared artefactual signal among brain regions (Chen et al., 2012; Power et al., 2014).

(R2Q6) Finally, in recommendations, I would suggest that you drill down a bit on how much these effects may influence other studies that likely have a distribution of time of day or a fixed time of day for their studies - evenly distributed across comparison populations. It's not clear to me that it is something we really need to worry about unless we are studying one population in the morning and one in the evening. It may have a more significant impact on attempts to characterize individuals (i.e. fingerprinting). More discussion on these nuances would be appropriate.

We thank the reviewer for pushing us to improve this part of our discussion. We have completely re-written the section to provide a more nuanced view of the potential impact of time of day on different types of studies in the field.

Section 4.10 of Discussion

> In this paper we have shown systematic time of day effects in the HCP dataset on resting state fMRI measures that are greater in magnitude to behavioural associations (e.g. fluid intelligence), consistent in both between- and within-subject analyses, present following regression of respiratory variation, and replicable across two sessions. Leaving aside the important challenge of understanding their underlying mechanisms, another important question to address is which studies are most likely to be affected by time of day and to what extent.
>
> Large-scale studies such as the HCP are particularly susceptible to time of day effects because they are more likely to have subjects scanned over a wide range of times in order to facilitate data collection. Although we found time of day effects to be modest in absolute terms, explaining less than 4% of the variance in GS fluctuation or RSFC, these were still comparable or stronger than most behavioural – RSFC associations reported in fMRI studies. Thus, group-level correction for time of day could potentially help avoid masking out or introducing spurious behavioural – RSFC associations in other similar large-scale studies with varying times of day of scans.
>
> In contrast, most small-scale studies will not be affected as these often tend to scan subjects in fixed timeslots. Even studies where subjects are scanned several hours apart are unlikely to be drastically impacted based on the fact that it takes around 9 hours for time of day effects to reach a similar magnitude as run-effects on GS fluctuation (see Figure 1E). Notable exceptions are longitudinal studies that seek to examine neural changes associated with same-day skill acquisition, as recently shown by Steel and colleauges (Steel et al., 2019). In general, all studies should avoid a situation where there is a non-random assignment of an experimental group or condition to a specific time of day. Group-level correction for time of day is unlikely to be useful in small-scale studies as they may lack statistical power to reliable estimate time of day effects.
>
> We recommend reporting time of day of fMRI scans and other experimental protocols and measurements. Even if all subjects are scanned at the same timeslot within a particular study, reporting time of day could help account for between-study variation in results and potentially even failed replications. Meta-analyses could then be leveraged to explore how time of day affects various regions, networks and tasks across different domains of the literature.

Reviewer #3:

(R3Q1) General comments: In this study the authors look at the effect of time of day on the magnitude of resting-state fMRI global signal fluctuations and connectivity. They find that there is a decrease in both global signal magnitude and resting-state connectivity. While somewhat interesting, the effects are rather weak, accounting for less than 4% of the variance in the data. The title, abstract, and main text need to be significantly modified to acknowledge how weak this effect especially in comparison to the much stronger relations with other factors such as arousal that have been shown in prior studies.

We thank the reviewer for the close read of the manuscript. We have made a number changes (in our responses to other comments cited below), which remove the suggestion that time of day effects might be greater than arousal or respiration and which present the size of the time of day effect in a more interpretable fashion.

In response to R3Q7 we have re-written the sentence in the Abstract which previously stated that our findings "challenge the prevailing notion that the brain's global signal mostly reflect arousal or physiological artefacts".

Abstract

> These findings reveal unexpected effects of time of day on global brain activity that are not easily explained by arousal or physiological artefacts.

In response to R2Q2 and R3Q8, we now explicitly state the magnitude of the decrease of GS fluctuation in terms of BOLD percent change in the Results section.

Section 3.1 of Results

> On average, the magnitude of GS fluctuation decreased from 0.22% to 0.17% (on average) between 9am to 9pm in both Sessions 1 and 2.

In response to R2Q6 and R3Q15 we have completely re-written our recommendations to provide a more nuanced discussion of the extent to which time of day confounds should be a cause for concern (Section 4.10)

Section 4.10 of Discussion

> In this paper we have shown systematic time of day effects in the HCP dataset on resting state fMRI measures that are greater in magnitude to behavioural associations (e.g. fluid intelligence), consistent in both between- and within-subject analyses, present following regression of respiratory variation, and replicable across two sessions. Leaving aside the important challenge of understanding their underlying mechanisms, another important question to address is which studies are most likely to be affected by time of day and to what extent.
>
> Large-scale studies such as the HCP are particularly susceptible to time of day effects because they are more likely to have subjects scanned over a wide range of times in order to facilitate data collection. Although we found time of day effects to be modest in absolute terms, explaining less than 4% of the variance in GS fluctuation or RSFC, these were still comparable or stronger than most behavioural – RSFC associations reported in fMRI studies. Thus, group-level correction for time of day could

potentially help avoid masking out or introducing spurious behavioural – RSFC associations in other similar large-scale studies with varying times of day of scans.

In contrast, most small-scale studies will not be affected as these often tend to scan subjects in fixed timeslots. Even studies where subjects are scanned several hours apart are unlikely to be drastically impacted based on the fact that it takes around 9 hours for time of day effects to reach a similar magnitude as run-effects on GS fluctuation (see Figure 1E). Notable exceptions are longitudinal studies that seek to examine neural changes associated with same-day skill acquisition, as recently shown by Steel and colleauges (Steel et al., 2018). In general, all studies should avoid a situation where there is a non-random assignment of an experimental group or condition to a specific time of day. Group-level correction for time of day is unlikely to be useful in small-scale studies as they may lack statistical power to reliable estimate time of day effects.

We recommend reporting time of day of fMRI scans and of other experimental protocols and measurements. Even if all subjects are scanned at the same timeslot within a particular study, reporting time of day could help account for between-study variation in results and potentially even failed replications. Meta-analyses could then be leveraged to explore how time of day affects various regions, networks and tasks across different domains of the literature.

(R3Q2) The study would also benefit from a consideration of other data that might shed light on the state of the subjects that are potentially related to arousal. For example, in the HCP dataset there are also task-based paradigms – an examination of the performance of the subjects on these tasks could provide some information on subject state.

We agree with the reviewer that exploring time of day effects on task paradigms would be interesting, but we think that this is beyond the scope of the current paper. However, we have added this suggestion to the discussion.

Section 4.11 of Discussion

There are many avenues to extend the current study. For example, it will be interesting to explore whether the same effects can be see during task-fMRI. In addition, Glasser and colleagues proposed the use of temporal ICA (Glasser et al., 2018) to decompose the fMRI data into multiple components, some of which appeared to reflect “global” artefacts, which can then be more selectively removed. It would be interesting to investigate how these global artifactual components might relate to time of day. Furthermore, some “global” components are present only during resting-fMRI, but not task-fMRI (Glasser et al., 2018). Thus, some of the effects we observe in this study might not appear in task-fMRI.

(R3Q3) There should also be some consideration of how the fact that subjects were engaged in a lengthy fMRI experiment might have affected the diurnal variations. An fMRI experiment is a rather an unusual activity to partake in and it reasonable to expect that this could lead to a deviation from the “usual” variations. For example, anticipation of the exam might grow over the course of the day. This is an important point to address because the authors assume throughout their paper that the subjects are experiencing a “typical”

decrease in arousal over the course of the day – this assumption gives rise to the "paradoxical" observation. However, this assumption is not adequately supported given the special circumstances involved in performing an experiment.

The reviewer raises a valid point regarding the potential impact of the study itself on our measures of subjects' GS fluctuations, which we now acknowledge in the limitations section (as quoted below):

Section 4.9 of Discussion

> Finally, the experience itself of taking part in an extensive study and of being scanned could have resulted in deviations in subjects' brain states from typical diurnal fluctuations of arousal. For example, participants scanned in the morning versus evening could have experienced differential levels of arousal or stress (e.g. due to the buildup of anticipation to being scanned)

(R3Q4) The paper would benefit from a more thorough treatment of the considerable amount of scatter in the data. This may be partly addressed through better plotting of the data (see below).

We thank the reviewer for the many suggestions, which we have tried our best to address. Please see our responses to your specific comments below.

(R3Q5) On a related note, there also needs to be a more detailed consideration of how large global signal fluctuation values may be driving the least squares fit and the windowed means. For example in Figure 1a,b there seem to be both fairly long tails of the GS values at each time of day and a great deal of temporal variability in the behavior of the tails. It would be good to verify that the scans with these large GS values exhibit reasonable behavior and to also examine the effects using robust estimators. Furthermore, an examination of the residuals is needed to determine the extent to which heteroscedastic effects are affecting the fit.

The reviewer raises a valid concern regarding the distribution of the data. Indeed visual inspection of the residuals suggested slight heteroscedasticity, driven by a greater variance of residuals at earlier than later times of day. We carried out two separate control analyses in order to assess the extent to which this could have affected our model fit (Figure S9).

First, we repeated our analysis of the relationship between time of day and GS fluctuation using robust regression with a Huber weighting function (Figure S9). In practice, robust regression is less affected by heteroscedasticity since it downweighs the influence of outlier observations. In our case, we found the fit from robust regression and from OLS to be highly consistent, thus suggesting that heteroscedasticity was not introducing substantial bias into our least squares fit. Second, we also computed a median quantile regression fit, which does not make any assumption of homoscedasticity. This also revealed a highly comparable model fit to OLS and to robust regression (Figure S9).

We also note that the range of GS fluctuation values reported in our analyses (including the high values GS fluctuation) are comparable to another paper which reported GS fluctuation values in terms of percent change (Liu et al,. 2017). Furthermore, the resting state scans included in this analysis have passed through the HCP's quality checking and preprocessing pipelines. In addition we have excluded runs with excessive levels of head motion (> 50% of

outlier frames), while we also show that time of day effects remain present after regressing out effects of respiration (Figure 3; Figure S4). As we discuss in the manuscript, high levels of GS fluctuation values could be driven by a range of physiological (heart rate, respiration) and neural factors (arousal, sleep), in addition to head motion and hardware artifacts. However, without a clear rationale, we do not feel confident assuming that high GS values should necessarily be excluded or down-weighted. However, our control analyses above (Figure S9) suggest that even if we did remove or down-weight outliers, our model fit would remain largely unchanged.

We have updated the paper as follows:

Section 3.8 of Results

> We also considered the possibility that the observed linear fit between GS fluctuation and time of day could have been influenced by heteroscedasticity of the data. Therefore we carried out two additional types of regression in addition to OLS, which do not assume homoscedasticity: robust-regression and quantile regression. We found the line of best fit to be highly similar accross all three types of regression a shown in Figure S9.

Section 5.7 of Materials & Methods

> We compared the line of best fit for the effect of time of day on GS fluctuation using three types of regression analyses: OLS, robust regression and quantile regression. The rationale was to observe the potential effect of heteroscedasticty, which should only influence OLS fit but not the other two approaches. We repeated this for both between-subject and within-subject analyses. OLS and robust-regression were computed using Scipy in Python, while robust-regression was run in MATLAB (robustfit) with Huber-weighting using the default tuning constant.

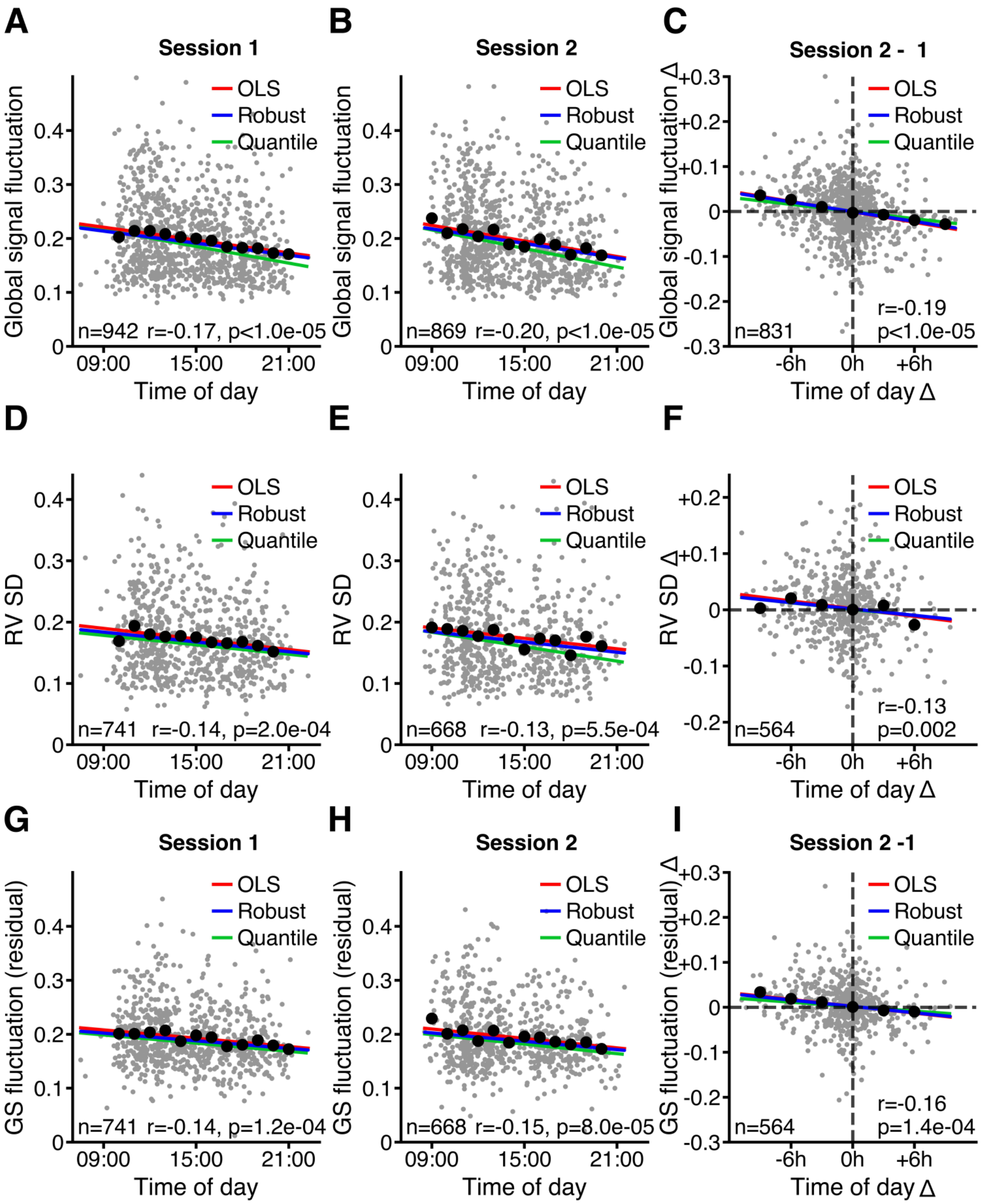

Figure S9. Scatterplots showing (A-C) effects of time of day on GS fluctuation, (D-E) effects of time of day on GS fluctuation on respiratory variation and (G-I) effects of time of day on GS fluctuation on GS fluctuation after controlling for respiratory variation. Lines of best fit were computed using three different methods: ordinary least squares regression (red), robust regression (blue) and quantile regression (green). Robust-regression and quantile regression were chosen because these two approaches are less susceptible to heteroscedasticity than OLS. The r and p-values shown are from the OLS regression. The same scatterplots are also presented in Figures 1, 3, S2, S3, S4 and S5.

(R3Q6) Overall, a better analysis of outlier, leverage, and influence effects is needed. For example, in Figure 1C. most of the data appears to be centered about 0h, but the line is probably overly influenced by the relatively fewer observations at the extreme ends. Indeed if one to provide standard error estimates of the windowed values, then the standard error would be quite small around 0h and increase greatly as one goes to the extremes.

As suggested by the reviewer, we have computed the standard error of the mean for the windowed values. We report these in Figure S8 as the standard error values were generally small and hard to see in the scatterplots.

The reviewer's observation is correct that the standard error is greater for the within-subject analyses at the tail ends, i.e. there were relatively fewer subjects with say a 9 hour difference between their scans on the two days than subjects that were scanned at around the same time of day. We already partially mitigate this concern in Figure 1 by computing windows over 3-hour periods for the within subject-analyses rather than 1-hour periods that are used for the between-subject analyses. Furthermore, the robust regression and quantile regression analyses presented in Figure S9 suggest that the model-fit was not substantially affected by greater variance at the tail-ends.

Finally, we note that the model fit for the within-subject analysis is corroborated by the model fit in the between-subject analyses for both Session 1 and for Session 2, where standard error was fairly stable across different times of day. The consistency across these different analyses should alleviate some of the concern stemming from the unequal number of observations at the tail-ends of the within-subject analysis.

We have updated the manuscript as follows:

Section 3.8 of Results

> We first visualised the standard error of the windowed means, which suggested similar spread of GS fluctuation across different times of day in between-subject analyses (Figure S8). In within-subject analyses, the distribution was more dissimilar across times of day, with early and later times exhibiting greater standard error due to presence of fewer data points than times around midday (Figure S8).

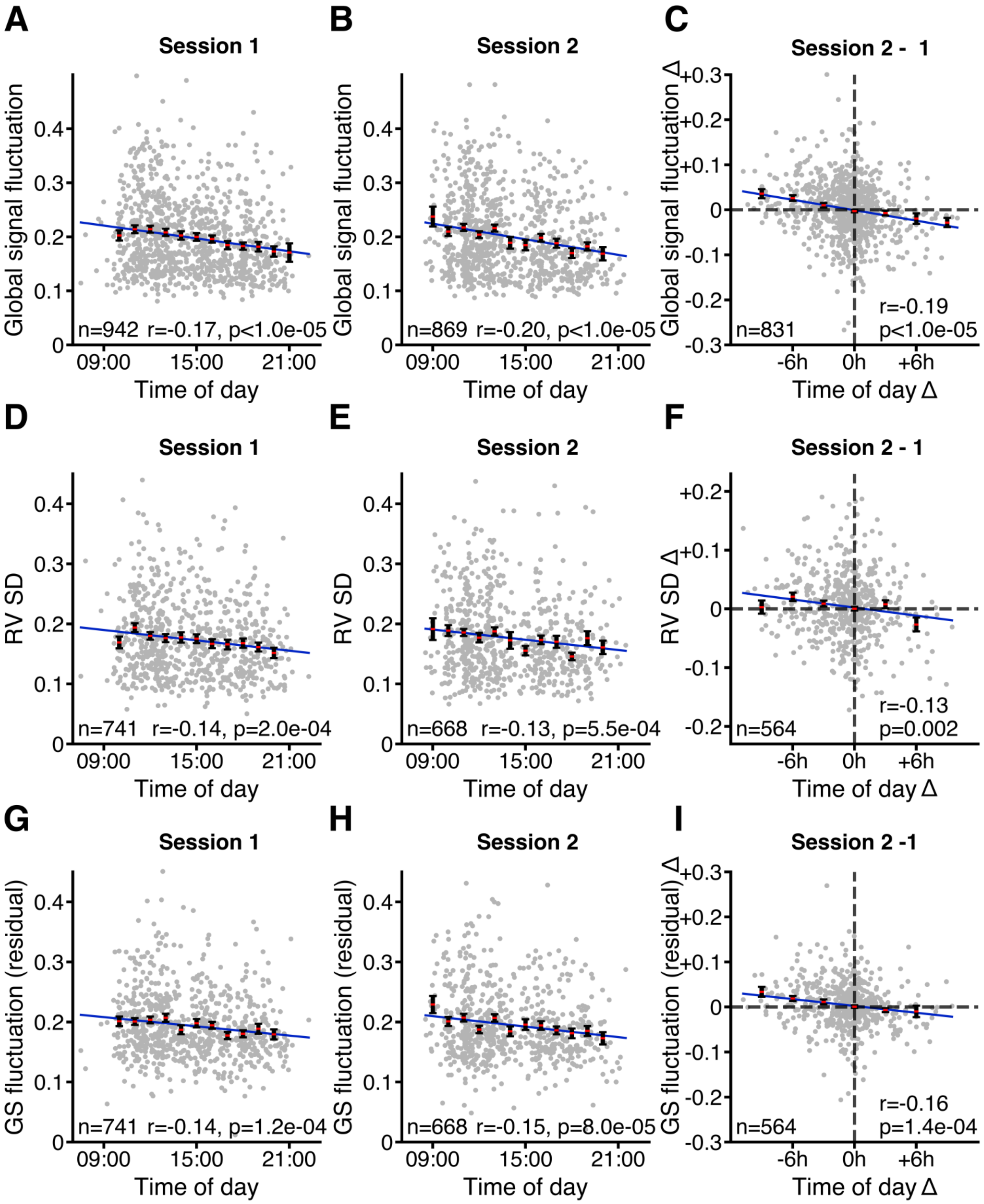

Figure S8. Scatterplots showing (A-C) effects of time of day on GS fluctuation, (D-E) effects of time of day on respiratory variation and (G-I) effects of time of day on GS fluctuation after controlling for respiratory variation. Error bars show standard error of hourly windowed means. These scatterplots are presented and described in more detail in Figures 1, 3, S2, S3 and S4, without the windowed standard error bars.

(R3Q7) Additional comments. It is misleading to say these results "challenge the prevailing

notion that the brain's global signal reflect mostly arousal and physiological artifacts" – while the authors show a rather weak effect, other studies show a very strong and robust relation between global signal and arousal and physiological artifacts. For example, the effects are particularly strong and repeatable as the subjects go from wakefulnesss to sleep.

We agree with the reviewer and have rephrased the sentence (see below).

Abstract

> These findings reveal unexpected effects of time of day on global brain activity that are not easily explained by arousal or physiological artefacts.

(R3Q8) It is also misleading in the abstract to contrast the 22% decrease in the global signal with a 1 to 3% evoked BOLD responses, especially given the weak nature of the effect. There is enough sensitivity to detect the task-related response in a single scan and voxel, whereas the observed GS effect is only weakly seen even given a very large sample size. Also, the GS magnitude is on the order 0.2% so a 22% change corresponds to a change of only 0.04% in BOLD percent change units. This gives a more meaningful sense of the change.

We thank the reviewer (as well as Reviewer 1) for challenging us on this point. We agree and therefore have removed this sentence from the abstract.

In addition we have also rephrased a sentence in the Results to express GS fluctuation in units of BOLD percent change as per the reviewer's advice:

Section 3.1 of Results

> From 9am to 9pm, the magnitude of GS fluctuation decreased from 0.22% of mean BOLD signal fluctuation to 0.17% (on average) in both Sessions 1 and 2.

Section 5.2 of Materials & Methods

> Global signal fluctuation was always plotted in units of BOLD percent signal change. This was calculated by dividing each subject's global signal fluctuation scores by 10,000 and multiplied by 100, given that each resting state fMRI run of each subject had originally undergone grand-mean scaling to a value of 10,000 during preprocessing.

(R3Q9) For the scatter plots, it would be useful to provide some indication of the density of the points – for example, using something like scatplot or dscatter in MATLAB.

As requested we have produced visualisations of the density of the scatterplots. We present these in Figure S1 for the sake of maintaining the simplicity of Figures 1 and 3 in the main text.

Section 3.1 of Results

> Subjects exhibited substantial between-subject variation in GS fluctuation at each time of day (see Figure S1 for a visualisation of scatterpoint density).

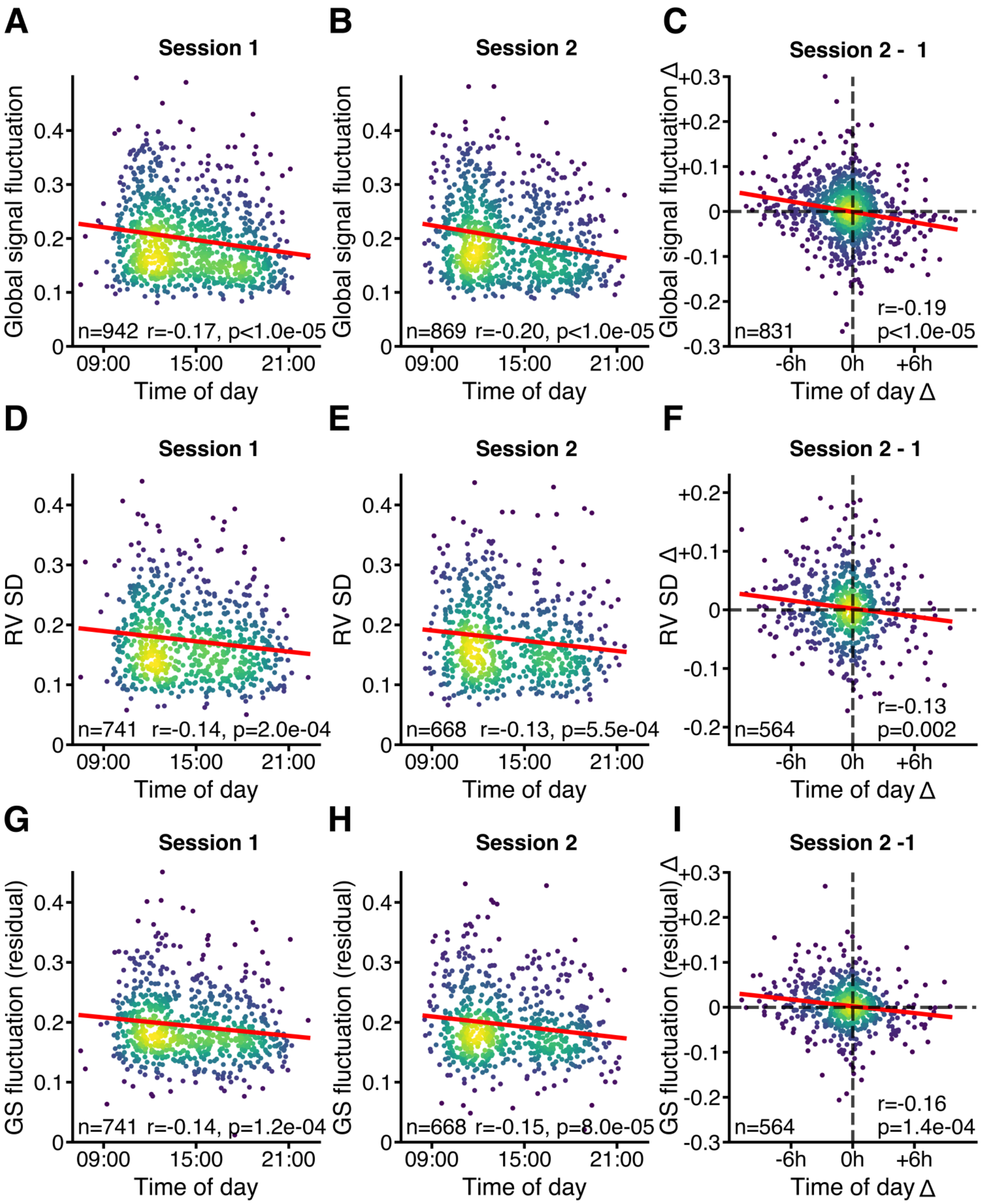

Figure S1. Scatterplots showing (A-C) effects of time of day on GS fluctuation, (D-E) effects of time of day on respiratory variation and (G-I) effects of time of day on GS fluctuation after controlling for respiratory variation with colour-coding of data point density. High density of data points around 12:30 pm is consistent with the planned timing of resting state scans based on the HCP study protocol (HCP Reference Manual - 1200 Subjects Release; Page 33). These same results are presented and described in more detail in Figures 1, 3, S3, S4, S8 and S9 without colour-coding of data point density.

(R3Q10) Confidence intervals for the regression plots should also be provided.

We have added confidence intervals for our plots (see Figure 1, Figure 3, Figure S2 and Figure S3).

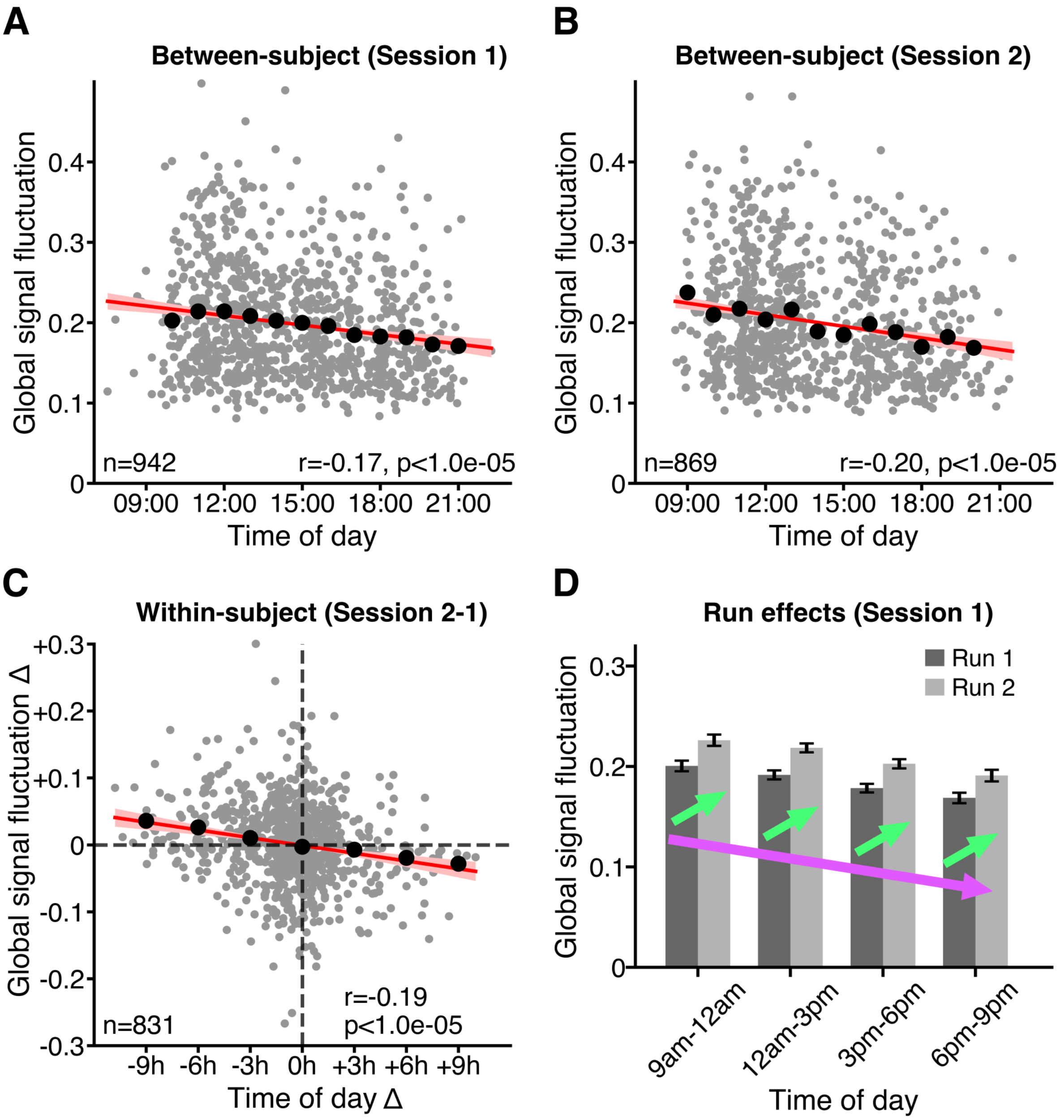

Figure 1. The brain's global signal (GS) fluctuation (i.e., standard deviation of global signal) decreases with time of day. (A) Between-subject variation in GS fluctuation as a function of time of day in Session 1. **(B)** Between-subject variation in GS fluctuation as a function of time of day in Session 2. **(C)** Within-subject variation in GS fluctuation Δ as a function of time of day Δ, where Δ denotes difference between Session 2 and Session 1. Grey dots denote individual subjects. Black dots show mean of GS fluctuation in (A, B) hourly, or (C) 3-hourly time windows. Line of best fit (red) was calculated based on data from all subjects in each plot. Confidence interval is shown in light red. R values denote Pearson r correlation coefficient. P values were derived from 100,000 permutations, while keeping family structure intact (Winkler et al., 2015). **(D)** GS fluctuation is elevated in Run 2 compared with Run 1 despite downward shift in GS fluctuation as a function of time of day.

Bar plots denote mean GS fluctuation across subjects within 3-hourly time windows for each run. Error bars denote standard error of the mean. Two opposing effects are observable: a fast increase in GS fluctuation on the scale of minutes, i.e. run effect, (green arrows), superimposed on a downward drift of GS fluctuation occurring on the scale of hours, i.e. time of day effect (violet arrow).

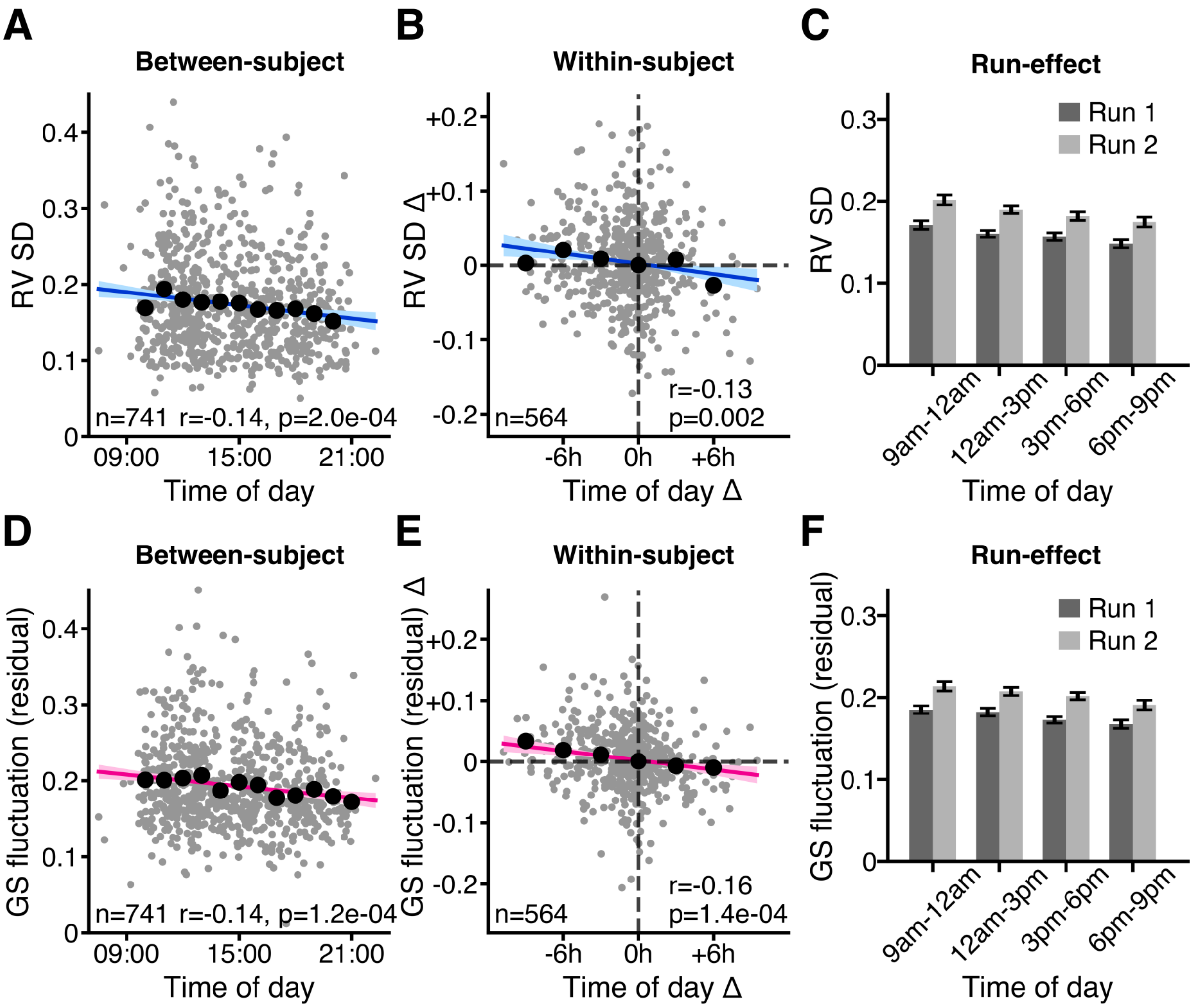

Figure 3. Negative association between time of day and GS fluctuation remains significant after controlling for respiratory variation. (A) Between-subject variation, **(B)** within-subject variation, and **(C)** run-effects of respiratory variation standard deviation (RV SD) as a function of time of day. **(D)** Between-subject variation, **(E)** within-subject variation and **(F)** run-effects of GS fluctuation residual (after regressing RV SD) as a function of time of day. Same as in Figure 1, within-subject effects were computed by taking the difference (Δ) for each variable between Session 2 and Session 1. Grey dots denote individual subjects. Black dots denote mean of GS fluctuation in hourly (**A, D**), or 3-hourly (**B, E**) time windows. Confidence interval is shown in light blue or light violet. r values denote Pearson r correlation coefficients. P values were derived from 100,000 permutations while keeping family structure intact (Winkler et al., 2015). SD refers to standard deviation. This figure shows the results for Session 1 (see Figure S3 for Session 2).

(R3Q11) Standard errors should be displayed for the windowed estimates.

Due to the small size of the standard error across different times of day, it was difficult to visualise these effectively within our scatterplots. However, given the reviewer's request, we show these in supplementary Figure S8 (as also mentioned in our response to R3Q6).

In addition, we note that standard error for 3-hour windowed estimates is already present in the main text in Figure 1D, Figure 3C and Figure 3F of our original (and revised) submission.

(R3Q12) It would be useful to demonstrate the combined dependence of GS magnitude on both time of day and RV SD. For example a 3D scatter plot of the data with time of day and RV as x and y axes and GS magnitude as z-axis may be interesting.

In response to the reviewer's suggestion we have produced colour-coded scatterplots of the GS fluctuation - time of day association, where colours denote the magnitude of RV SD (Figure S4). Not surprisingly, the colours reveal a visual gradient where subjects who have greater RV SD tend to exhibit greater GS fluctuation (brighter colours; Figure S4A-B). Or in the case of within-subject analyses, those with a greater within-subject difference in RV SD, exhibit a greater within-subject difference in GS fluctuation (fuller colours; Figure S4C). As one would expect, these apparent gradients vanish following group-level regression of RV SD from the GS fluctuation (Figure S4D-F).

Section 3.4 of Results

> The negative correlation between time of day and GS fluctuation residual remained significant, and only slightly attenuated in magnitude (Figure 3D-F and Figure S3; Figure S4).

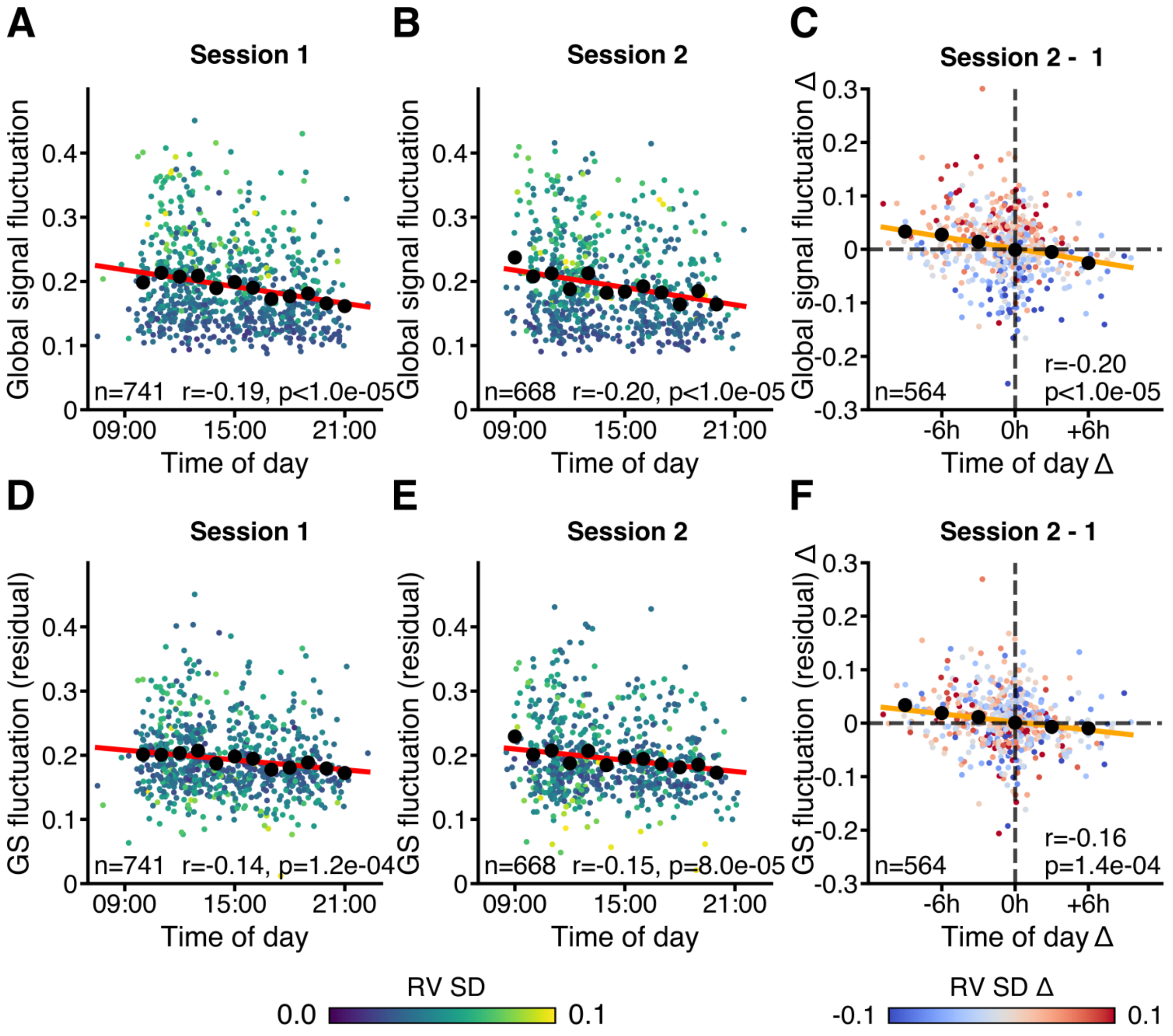

Figure S4. Effects of time of day and of respiratory variation (RV SD) on global signal fluctuation (GS fluctuation). (A, B) Subjects with greater RV SD (brighter dots) and scanned earlier in the day are more likely to exhibit greater GS fluctuation than those with lower RV SD (darker dots) and those scanned later in the day **(C)** Subjects scanned a longer duration apart on the two sessions (greater time of day Δ) are more likely to exhibit a greater between-session difference in GS fluctuation (greater GS fluctuation Δ) and in RV SD (greater RV SD Δ). Subjects with greater RV SD on Session 2 are denoted in red, while those with higher RVSD on Session 1 are denoted in blue. **(D-F)** As expected, statistically controlling for the effects of RV SD on GS fluctuation via group-level regression eliminates the apparent visual gradient pattern along the y-axis reflecting the systematic contribution of RV SD to GS fluctuation. These results are also presented in Figures 3 and S2 without colour coding of RV SD.

(R3Q13) It would be more meaningful to report the GS magnitude in terms of percent change BOLD signal. Presumably, the preprocessed data has used the HCP preprocessing that scales each 4D volume to a grand mean value of 10,000. If this is the case, then the global signal fluctuation magnitudes reported in Figure 1 are on the order of 0.2% which is consistent with prior work.

We agree and have done so in the revised manuscript. See our response to R3Q8.

(R3Q14) P. 8 – it is stated that the correction for multiple comparisons (when considering different measures) is explained in the Methods, but this does not appear to be the case. The multiple comparisons explanation in Methods appears to be for the regional maps.

We thank the reviewer for spotting this omission. We have now added the description of our multiple comparisons controls in two sections where it was missing in our initial submission.

Section 5.1.5 of Materials & Methods

> False discovery rate (FDR) correction was applied at $q < 0.05$ to all 1815 statistical tests conducted in this manuscript. These 1815 statistical tests include analyses presented across Sections 3.1 to 3.7.

Section 5.3.1 of Materials & Methods

> We repeated the FDR-correction at two additional thresholds, thus allowing us to denote the level of significance for each correlation in Figure 2 (* $q < 0.05$; ** $q < 0.01$; *** $q < 0.001$).

(R3Q15) Given the weak observed effects, the recommendations in section 4.9 seem a bit of an overreach – if the authors are to make such recommendations, it would be useful for them to provide an estimate of how much time of day would affect the conclusions of a typical fMRI study, which in general have much smaller sample sizes.

We have now entirely re-written our recommendations to provide a more balanced view on how it would impact various types of studies in the field. Please see our response to R3Q1.
