# Supplementary material for: Time of day is associated with paradoxical reductions in global signal fluctuation and functional connectivity
Source: PLoS Biol. 2020 Feb 18;18(2):e3000602. doi: 10.1371/journal.pbio.3000602 (PMC7028250; doi:10.1371/journal.pbio.3000602)
Supplement: Attachment — Submitted filename: Orban_2020_final_revision_Reply_to_Reviewers.pdf [file pbio.3000602.s014.pdf]

Reply to reviewers PBIOLOGY-D-19-01472R1 "Time of day is associated with paradoxical reductions in global signal fluctuation and functional connectivity"

We thank the reviewers for a second reading of our manuscript and for the additional suggestions. We have addressed these as shown below. Please find our responses (in blue) to the reviewers' comments below (in italics). For convenience, changes to the manuscript are quoted verbatim (normal font) when appropriate.

Reviewer #1

R1Q1 Matthew F. Glasser: The authors have addressed my prior concerns. I would request a minor tweak of the future section to read "There are many avenues to extend the current study. For example, it will be interesting to explore whether the same effects can be seen during task-fMRI. In addition, Glasser and colleagues proposed the use of temporal ICA (Glasser et al., 2018) to decompose the fMRI data into multiple components, some of which appear to reflect "global" artefacts, which can then be more selectively removed, and some of which may relate to neural signals for arousal or eyes open versus closed. It would be interesting to investigate how these distinct global components might relate to time of day. Furthermore, some of these "global" components are present only during resting-fMRI, but not task-fMRI (Glasser et al., 2018). Thus, some of the effects we observe in this study might not appear in task-fMRI."

We have updated the following passage as requested.

Section 4.11 of Discussion

> There are many avenues to extend the current study. For example, it will be interesting to explore whether the same effects can be seen during task-fMRI. In addition, Glasser and colleagues proposed the use of temporal ICA (Glasser et al., 2018) to decompose the fMRI data into multiple components, some of which appear to reflect "global" artefacts, which can then be more selectively removed and some of which may relate to neural signals for arousal or eyes open versus closed. It would be interesting to investigate how these distinct "global" components might relate to time of day. Furthermore, some "global" components are present only during resting-fMRI, but not task-fMRI (Glasser et al., 2018). Thus, some of the effects we observe in this study might not appear in task-fMRI.

We have also added a URL where the list of subjects who passed our QC of the physiological data can be freely accessed.

Section 5.4.2 of Materials and Methods

> The list of subjects who passed visual quality screening of their pulse and respiratory data is publicly available at the GitHub repository maintained by the Computational Brain Imaging Group (https://github.com/ThomasYeoLab/CBIG/tree/master/stable_projects/preprocessing/Orban2020_tod/data_release).

Reviewer #3

The authors have made substantial revisions that largely address the prior concerns. I just have two remaining minor comments.

R3Q1: In the abstract, the authors now state: "These findings reveal unexpected effects of time of day on global brain activity that are not easily explained by arousal or physiological artefacts." However, I think this is still misleading as the authors did not actually make any measurements of arousal state. I think what they mean to say is the "expected arousal" state. As noted in the prior comments and as acknowledged by the authors in the revised work, the actual arousal state may have differed from the expected arousal state due to a number of factors.

We agree with the reviewer's point and have further refined this sentence in the abstract as suggested.

Abstract:

> These findings reveal time of day effects on global brain activity that are not easily explained by expected arousal state or physiological artefacts.

R3Q2: For Figure S1, please add a colorbar to describe the color scheme used.

We have now added colourbars to Figure S1.

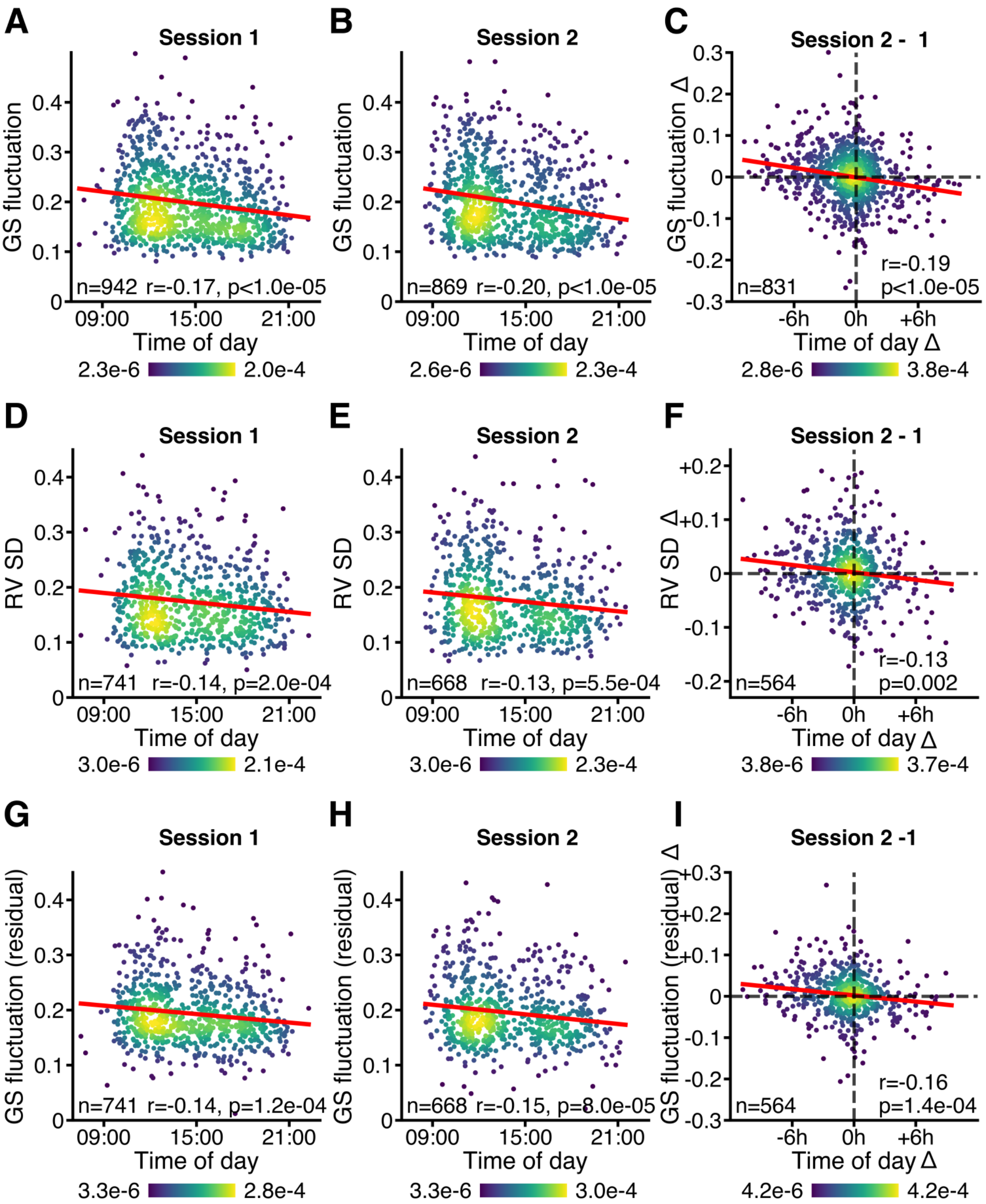

Fig S1. Scatterplots showing (A-C) effects of time of day on GS fluctuation, (D-E) effects of time of day on respiratory variation and (G-I) effects of time of day on GS fluctuation after controlling for respiratory variation with colour-coding of data point density. High density of data points around 12:30 pm is consistent with the planned timing of resting state scans based on the HCP study protocol (HCP Reference Manual - 1200 Subjects Release; Page 33). These same results are presented and described in more detail in Fig 1, 3, S3, S4, S8 and S9 without colour-coding of data point density.
