# Supplementary figures and images for: Correction: Time of day is associated with paradoxical reductions in global signal fluctuation and functional connectivity
Source: PLoS Biol. 2021 May 18;19(5):e3001258. doi: 10.1371/journal.pbio.3001258 (PMC8130927; doi:10.1371/journal.pbio.3001258)

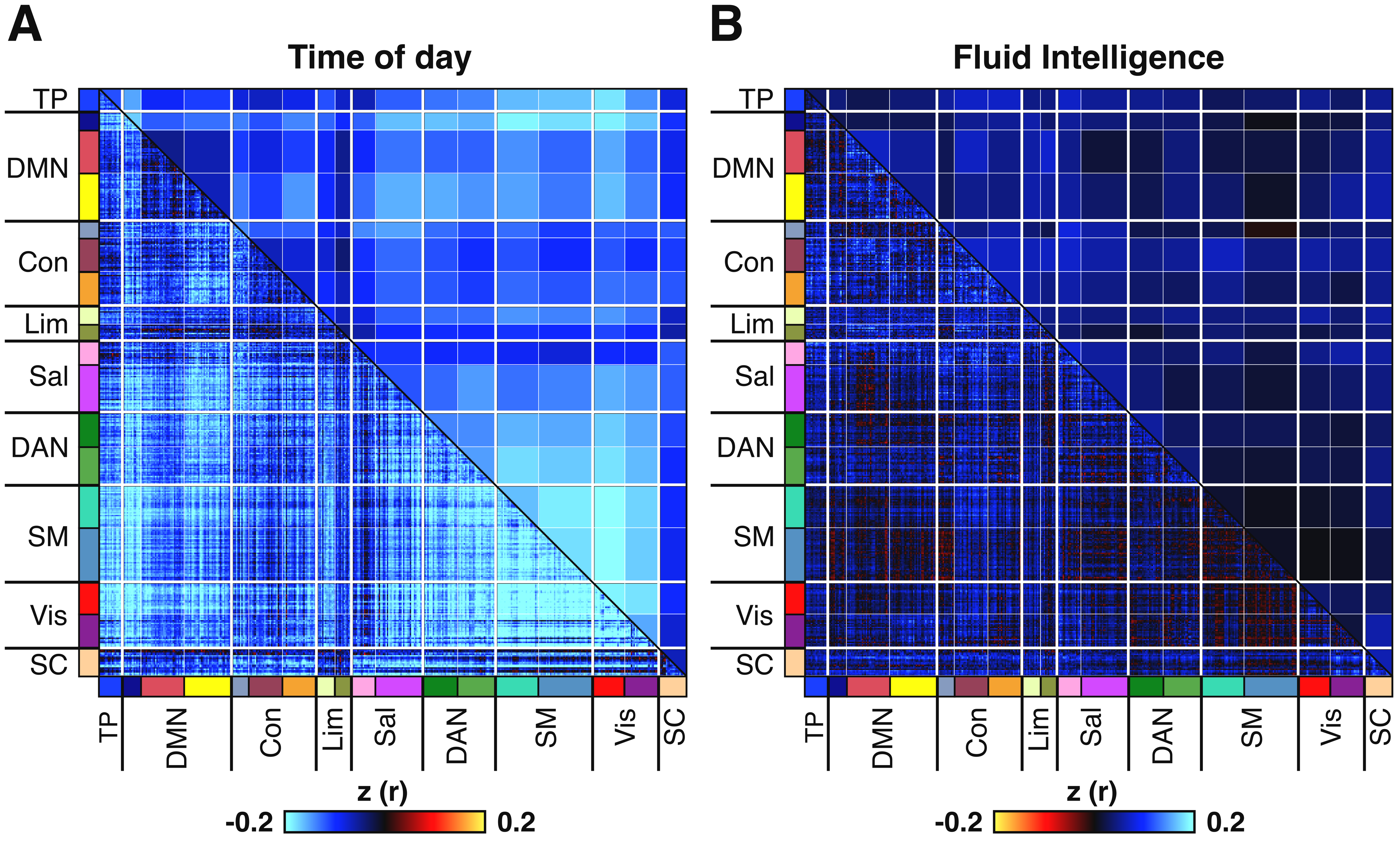

Supplement: S5 Fig — (A) Correlation between time of day and RSFC across participants. (B) Correlation between fluid intelligence and RSFC across participants. Colours in lower triangular of correlation matrix denote z-transformed Pearson r correlation coefficients. Colours in the upper triangular denote z-transformed r values from the lower triangular averaged within network pairs. Colours on label axes denote correspondence of 419 regions to 17 large-scale cortical networks and to SC. Median absolute z values computed over the lower triangular were higher for time of day (0.13) than for fluid intelligence (0.04). Time of day–RSFC effects were significant, whereas RSFC–fluid intelligence effects were not significant for session 2, as assessed by network-based statistics (FDR-corrected at q < 0.05). Note that the colour scale for the fluid intelligence–RSFC effects was inverted to facilitate visual comparison with time of day effects. For session 1 results, see Fig 6 in the main text. FDR, false discovery rate; RSFC, resting state functional connectivity; SC, subcortex. (TIF) [file pbio.3001258.s001.tif]

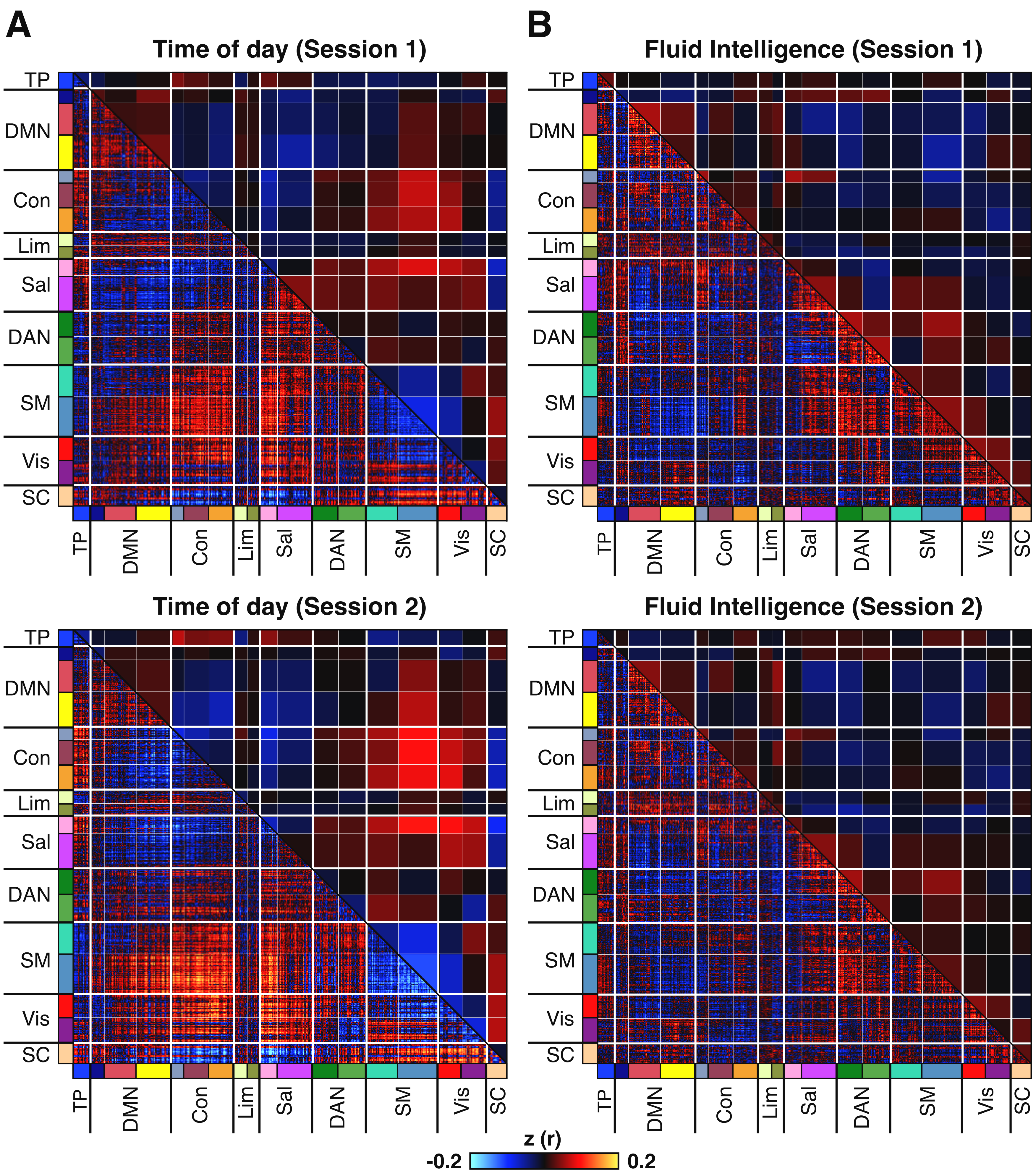

Supplement: S7 Fig — (A) Correlation between time of day and RSFC across participants. (B) Correlation between fluid intelligence and RSFC across participants. Levels of correlation are visibly stronger between time of day and RSFC than between fluid intelligence and RSFC. Colours in lower triangular of correlation matrix denote z-transformed Pearson r correlation coefficients. Colours in the upper triangular denote z values from the lower triangular averaged within network pairs. Colours on label axes denote correspondence of 419 regions to 17 large-scale cortical networks and to SC. Time of day–RSFC effects were significant in both sessions as assessed by network-based statistics (FDR-corrected at q < 0.05), whereas fluid intelligence–RSFC effects were significant only in session 1. FDR, false discovery rate; RSFC, resting-state functional connectivity; SC, subcortex. (TIF) [file pbio.3001258.s002.tif]
